# Probing Intermolecular H-Bonding Interactions in Cyanuric Acid Networks: Quenching of the N *K*-Edge Sigma Resonances

**DOI:** 10.1021/acs.jpca.2c04517

**Published:** 2022-09-28

**Authors:** Valeria Lanzilotto, Daniele Toffoli, Elisa Bernes, Mauro Stener, Elisa Viola, Albano Cossaro, Roberto Costantini, Cesare Grazioli, Roberta Totani, Giovanna Fronzoni

**Affiliations:** † Department of Chemistry, Sapienza Università di Roma, P. le A. Moro 5, Roma, 00185, Italy; ‡ Department of Chemical and Pharmaceutical Sciences, University of Trieste, 34127 Trieste, Italy; § IOM-CNR, Istituto Officina dei Materiali-CNR, S.S.14, Km 163.5, 34149 Trieste, Italy; ∥ ISM-CNR, Istituto Struttura della Materia-CNR, LD2 Unit, S.S. 14, Km 163.5, 34149 Trieste, Italy

## Abstract

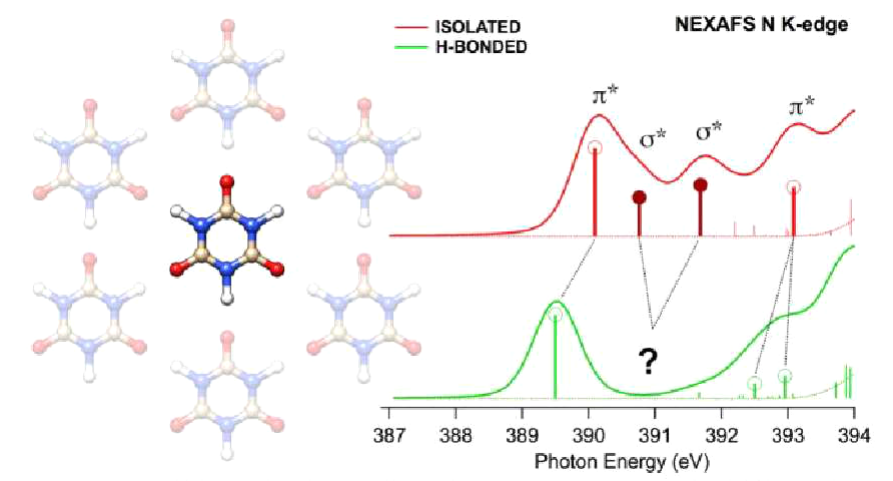

The electronic characterization of the cyanuric acid both in gas phase and when embedded within an H-bonded scheme forming a monolayer on the Au(111) surface has been performed by means of X-ray Photoelectron Spectroscopy (XPS) and Near Edge X-ray Absorption Fine Structure (NEXAFS) spectroscopy. The experimental spectra at the N, O, and C *K*-edges have been assigned with the support of DFT calculations, and the combination between theory and experiment has allowed to us investigate the effect of the H-bonding intermolecular interaction on the spectra. In particular, the H-bond formation in the monolayer leads to a quenching of the N 1s NEXAFS resonances associated with transitions to the sigma empty orbitals localized on the N–H portion of the imide group. On the other hand, the π* empty states remain substantially unperturbed. From a computational point of view, it has been shown that the DFT-TP scheme is not able to describe the N 1s NEXAFS spectra of these systems, and the configuration mixing has to be included, through the TDDFT approach in conjunction with the range-separated XC CAM-B3LYP functional, to obtain a correct reproduction of the N 1s core spectra.

## Introduction

Molecular recognition via hydrogen bonding is at the basis of many different current applications, such as sensing^1,2^ and biosensing^3^ pharmaceutical and semiconducting cocrystals engineering^4,5^ and catalysis.^6^ Recently, the triple H-bonding scheme of cyanuric acid (CA) has gained particular interest for its implementation in chemical sensors suited for the detection of melamine (M), an emerging contaminant in milk, infant formula, and pet food.^7^ For instance, a colorimetric mechanism for melamine recognition has been developed by using CA functionalized gold nanoparticles (CA-AuNPs).^8^ The H-binding of the melamine molecules causes nanoparticle aggregation and, consequently, a dramatic red-shift of the characteristic AuNPs plasmon resonance. Similarly, a fluorescence “turn-on” detection mechanism has been realized with aggregation-induced emission-active materials bearing the CA functionality.^9^ Actually, sensing applications for melamine recognition takes inspiration from the large variety of *rosette*- and *tape*-type crystalline structures obtained, since the late 1980s, through the rational functionalization of both melamine and cyanuric acid.^10−13^ The H-bonded CA*M adduct has been also largely exploited in surface science to build-up highly ordered 2D networks and more complex 3D architectures. Since 2006, 2D arrays of CA*M have been studied on several surfaces (i.e., Ag/Si(111), Au(111), HOPG) by means of the scanning tunneling microscopy (STM) technique.^14−18^ Later on, extensive morphological investigations have been focusing on the characterization of 3D heterostructures, where the 2D CA*M network is used to drive the epitaxial growth of distinct organic layers (i.e., trimesic acid, terephthalic acid, PTCDA, or C60) aiming to realize novel layered materials with tunable optical and electronic properties.^19,20^ More recently, the H-bonded porous CA*M network has been successfully used as catalyst support for embedding metal phosphide nanoparticles, giving excellent electrocatalytic hydrogen evolution activities under strong acidic conditions.^21^

Despite the vast design of CA*M-based applications and surface science experiments, a detailed spectroscopic characterization of the CA*M triple H-bonding interaction is still missing. In this regard, only the melamine molecule has been studied by means of electron spectroscopies both as noninteracting system (gas phase) and when adsorbed in H-bonded networks on Au(111)^22^ or chemisorbed on Cu(111).^23,24^ In passing, previous works have employed NEXAFS spectroscopy to study the influence of intermolecular interactions, including hydrogen bonds formation, on the NEXAFS spectra of small organic molecules in gas-phase and in crystal structures.^25,26^

In order to reach a comprehensive description of the CA*M interaction, in this work we perform a propaedeutic characterization of the CA molecule both in the gas-phase and when embedded within an H-bonded scheme forming a monolayer.

**Figure 1:**
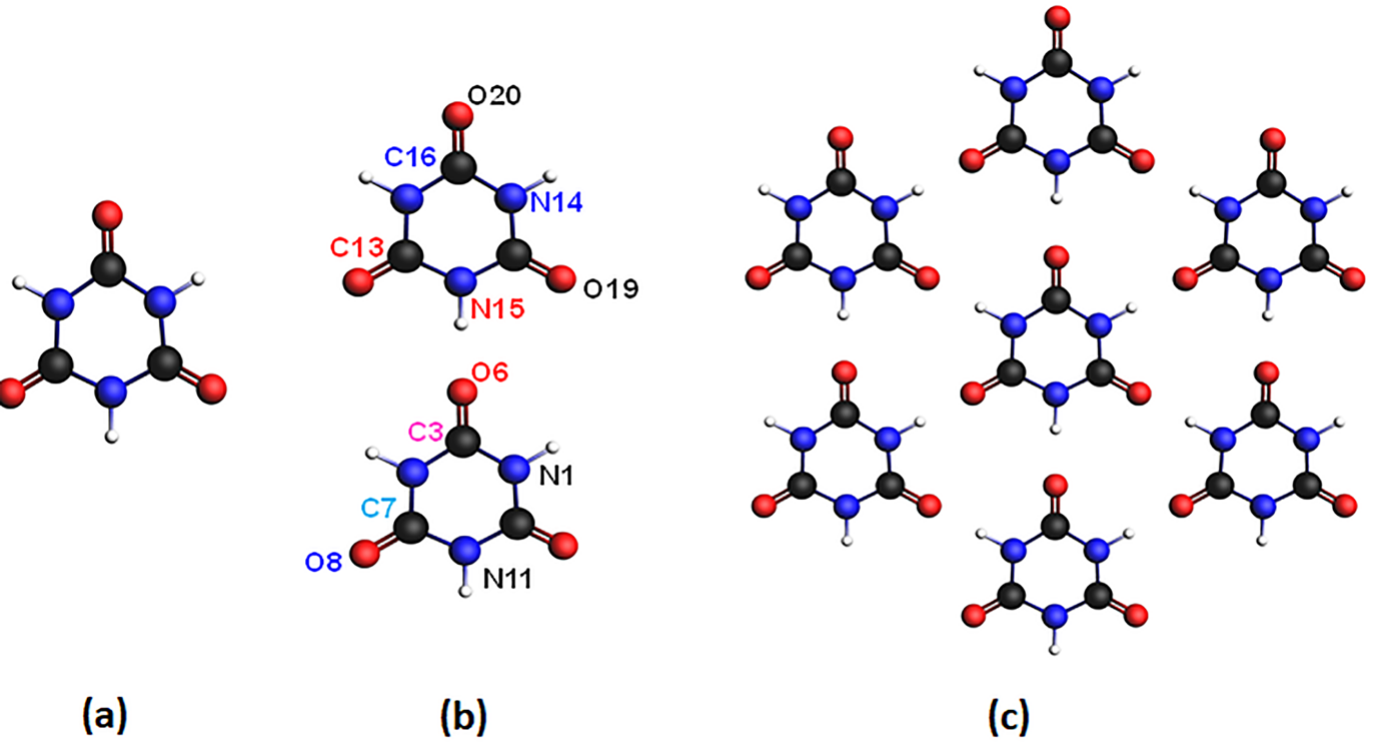
Chemical structure of (a) cyanuric acid (CA), (b) cyanuric acid dimer, and (c) H-bonded periodic structure (H–B model). C atoms are in black, N atoms in blue, O atoms in red, and H atoms in white. The labels on the CA dimer indicate the nonequivalent C, N, and O atoms.

Computationally, the effect of H-bond formation on the core spectra of CA (Figure 1a) has been investigated by considering the CA dimer (Figure 1b) and a periodic hexagonal arrangement of the CA monomers (Figure 1c). This latter, indicated as the H–B model, is considered as the model of the CA monolayer.^16^ In the H–B model, the central CA molecule is H-bonded to six neighboring molecules, acting both as a H-donor through the N atoms of the NH imide groups and as an H-acceptor via the carbonyl O atoms.

Experimentally, X-ray photoelectron (XPS) and absorption (NEXAFS) spectroscopy measurements have been performed in the gas phase as well as on a CA monolayer grown on the poorly reactive Au(111) surface.

We show in the following how fingerprints of the H-bonding interaction are successfully assessed by combining theory and experiments. In particular, the formation of H bonds in the monolayer leads to a quenching of the intensity of sigma transitions in the N *K*-edge NEXAFS.

## Experimental Section

Experiments were performed at the Elettra Synchrotron facility located in Trieste, Italy. Specifically, gas phase measurements were carried out at the GasPhase beamline.^27^ Cyanuric acid (CA) was purchased from Sigma-Aldrich (purity 98%) and sublimed from a stainless steel crucible kept at a temperature of 378 K (gas phase). The photoemission (PE) spectra were acquired with a VG-220i electron analyzers. The O 1s spectra were measured with photon energy of 628 eV (overall resolution 0.45 eV), while N and C 1s spectra were measured, respectively, with photon energies of 495 and 382 eV (overall resolution 0.28 eV). The calibration of the O and C 1s binding energy scale was made by the simultaneous acquisition of the O and C 1s spectra of the gaseous CO_2_ and aligning them to 541.3^28^ and 297.7 eV,^29^ respectively. The N 1s binding energy scale was calibrated by measuring the N 1s spectrum of the gaseous N_2_ and aligning the corresponding peak to 409.9 eV.^30^ The O, N and C *K*-edge NEXAFS spectra were measured in total-ion-yield mode with photon energy resolution set to 90, 55, and 50 meV, respectively. For the O and C *K*-edge, calibration of the photon energy scale was provided by simultaneous acquisition of the O and C *K*-edge NEXAFS spectra of the gaseous CO_2_ and locating the O 1s → π* and the C 1s → π* transitions at 535.4^31^ and 290.77 eV,^32^ respectively. Calibration of the N *K*-edge photon energy scale was performed by measuring the N *K*-edge NEXAFS spectrum of the gaseous N_2_ and locating the π* (ν′ = 1) resonance at 401.10 eV.^33^ The absorption intensity was normalized with the transmitted photon flux measured by a calibrated Si photodiode.

Solid state measurements were performed at the ANCHOR branchline^34^ of the ALOISA beamline. The Au(111) single crystal was cleaned by sputtering and annealing cycles. A monolayer of CA was prepared by sublimation of the CA powders from a quartz crucible held at ca. 423 K while keeping the substrate at room temperature. PE and NEXAFS spectra were acquired with a SPECS Phoibos 150 electron analyzer. The O 1s PE spectra were acquired with photon energy of 650 eV (overall resolution 0.2 eV), while the N 1s and C 1s PE spectra were measured with photon energy of 515 eV (overall resolution 0.15 eV). The energy scale was calibrated by aligning the Au 4f7/2 peak to the binding energy of 84.0 eV.^35^ All the PE spectra were measured with an emission angle of 35° and normal incidence. The NEXAFS spectra were acquired in Auger yield mode with photon energy resolution of 0.1 eV for C and N *K*-edges and of 0.2 eV for O *K*-edge. The spectra were collected with the electric field polarization of the light parallel (s-pol) and almost perpendicular (p-pol) to the surface plane. The photon energy was calibrated by using the Au 4f7/2 line measured with the last photon energy of each NEXAFS spectrum. The C *K*-edge spectra were normalized for the corresponding spectra acquired on the clean substrate. For O and N *K*-edge, the p-pol and s-pol were normalized in order to match pre-edge and postedge intensities.

## Theoretical and Computational Details

### a Geometry Optimization

A variable-cell relaxation algorithm was used to optimize the lattice parameters and the atomic coordinates of a free-standing 2D monolayer of CA H-bonded network at the DFT level within the plane-wave pseudopotential method as implemented in the QUANTUM ESPRESSO (QE) code suite.^36^ The overlayer is hexagonally closed-packed where each CA molecule interacts through six H bonds to its six neighbors.^16^ The exchange and correlation portion of the interaction energy between the electrons was described with the Perdew–Becke–Ernzerhof functional,^37^ including the D3 formulation of the van der Waals interactions.^38^ The ion-electron interaction was modeled by using ultrasoft pseudopotentials.^39^ A Monkhorst–Pack^40^ mesh of 4 × 4 × 1 points was used for the Brillouin zone integrations along with a 40 Ry kinetic energy cutoff to truncate the plane-wave basis and a corresponding cutoff of 400 Ry for the augmentation charge. A vacuum of 17 Å was used along the *z* direction to minimize the interaction between periodically repeated layers. The optimized value of the lattice parameters *a* and *b* of the hexagonal unit cell (*a* = *b* = 6.703 Å) compares with similar calculations in the literature.^16^ From this 2D structure we cut a finite model of a central CA molecule surrounded by six first-neighbors to be used in the subsequent calculation of the NEXAFS spectra.

The equilibrium geometry of the CA monomer in the gas phase was optimized both at the DFT B3LYP/aug-cc-pVTZ level through the Gaussian09 program^41^ and with the QE code suite^36^ by using the same plane wave basis set that has been adopted for the structural relaxation of the 2D CA overlayer. The CA monomer has been put inside a cubic box large enough to prevent interaction with periodic images and the first Brillouin zone was sampled at the Gamma point only. The two optimized geometries differ very little one another. A CA dimer was also carved out from the optimized 2D structure and used in the subsequent NEXAFS calculations without further optimizations.

### b XPS and NEXAFS Calculations

NEXAFS spectra at the C 1s, O 1s, and N 1s edges have been calculated with the transition potential (TP) DFT approach (DFT-TP)^42,43^ which is a cost-effective method for describing quite accurately relaxation effects that arise upon the formation of the core hole. In the TP framework, core-excitation energies are calculated as the energy difference of the final virtual and the core initial TP-MOs involved in the transition. Since this approach generally leads to a less attractive potential, the resulting absolute transition energies are usually too large compared to the experimental ones. To overcome this, we computed the IPs within the ΔSCF Kohn–Sham (ΔSCF) scheme, allowing a full relaxation of the ionized core hole, and then we shift the TP excitation energies by a value corresponding to the difference between the energy of the initial core-excited TP-MOs and IP^ΔKS^, where the IP^ΔKS^ is given by the difference between the energy of the *N* – 1 electronic configuration and that of the *N*-electron configuration. The energy of the 1 s^–1^ ionic state is calculated through a Kohn–Sham (KS) spin-polarized unrestricted scheme. A further rigid shift of the theoretical profiles has been applied for a better comparison with the measured spectra. The transition intensities are expressed as oscillator strength *f*_*i→f*_, which corresponds to the following equation for a randomly oriented (gas phase) molecule:
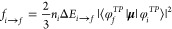
1For a fixed in space molecule, *f*_*i→f*_ reads:
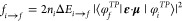
2Here the dipole moment integrals involve the initial and final TP MOs, *n*_*i*_ indicates the occupation number of the core orbital in the ground state, and **ε** is the polarization vector of the incident radiation.

NEXAFS spectra at the N *K*-edge were also calculated with linear-response time-dependent DFT (TDDFT)^44^ obtaining transition energies and intensities through the solution of the eigenvalue equation by using Davidson’s iterative algorithm.^45^ At the TDDFT level, the coupling between the single excited configurations is formally included. Since the core-excited states lie very high in energy, they can be computed efficiently by restricting the single excitation space to include only excitations from the subset of core orbitals^46^ according to the core–valence separation approximation introduced by Cederbaum et al.^47,48^

The calculations of C, N, and O *K*-edge NEXAFS spectra were carried out with the Amsterdam Density Functional (ADF) program.^49,50^ DFT-TP calculations were carried out within the generalized gradient approximation (GGA) PW86x Perdew^51^ and hybrid B3LYP^52−54^ xc potentials while TDDFT calculations were carried out with both B3LYP and the range-separated hybrid CAM-B3LYP^55^ xc potentials. For CA, all calculations were performed for its more stable *keto* isomer.^56^

An even-tempered quadruple-ζ with three polarization and three diffuse functions (ET-QZ3P-3DIFFUSE in the ADF database) Slater-type orbitals (STO) basis set has been employed for the core-excited C, N, and O atoms, to properly describe transitions to diffuse Rydberg states. A triple ζ polarized (TZP in the ADF database) basis set of STOs was adopted for the core orbitals of nonexcited C, N, and O atoms. In case of symmetry-equivalent atomic centers, a Frozen Core (FC) 1s basis set was employed to ensure the localization of the half core hole.

In the C, N and O *K*-edge NEXAFS spectra calculations of the CA dimer within the DFT-TP scheme, a separate computation of the excitation spectrum of each nonequivalent C, N, and O site was performed, and the partial contributions were summed to yield the total spectrum.

In each NEXAFS spectrum, the ionization thresholds are shown which are useful to separate the below-edge region, where a discrete orbital description is absolutely adequate, from the above-edge region, where only qualitative information can be extracted, as a consequence of the discretization of the continuum that is an artifact of the calculation.

The stick spectra have been convoluted by using Voigt and Gaussian profiles for transitions below and above the edge, respectively. For the Voigt profiles, a Lorentzian 0.2 eV and a Gaussian 0.7 eV broadening have been adopted for all the spectra, with the exception of the C *K*-edge curves, where a Gaussian 0.3 eV broadening better fits the experimental data. Gaussian functions with full-width-at-half-measure (fwhm) of 2.0 eV has been used above edge. A step function was introduced to account for the ionization edge.^64^

## Results and Discussion

In the following discussion, we will first consider the XPS spectra of CA in the gas-phase and adsorbed on Au(111) and their comparison with the theoretical results. We will then analyze the NEXAFS spectra at the N 1s, O 1s, and C 1s edges of the gas phase CA with the support of the theoretical calculations to provide an assignment of the experimental measurements. The influence of the hydrogen bond interaction will be investigated by comparing the calculated NEXAFS spectra of the gas-phase monomer with those of the H-bond model system; the theoretical results for the CA dimer (reported in the Supporting Information) will be used as a support to discuss the evolution of the spectral features for H-bonding systems with increasing number of CA units. The polarized experimental NEXAFS spectra of CA adsorbed on Au(111) will be finally compared with the theoretical spectra for the fixed in space H–B model in order to verify the reliability of the H–B model system in describing the CA 2D network on the surface and to identify possible effect of the surface–adsorbate interaction on the NEXAFS spectra.

The results obtained with DFT-TP combined with the B3LYP xc potential for N, O, and C *K*-edges will be presented, since the results computed with PW86x do not improve the description of the spectra.

## XPS Spectra

In order to evaluate how intermolecular H-bonding interactions affect the core level BEs, we exploited the Au(111) surface to grow a monolayer of CA. According to previous STM work,^16^ cyanuric acid is known to adsorb flat on Au(111) allowing the formation of H-bonding interactions of the type C=O–H–N, where each molecule acts both as H-donor and H-acceptor. The flat adsorption geometry is indeed verified by the NEXAFS dichroisms observed for all edges (see later). The experimental N 1s, O 1s and C 1s XPS spectra of CA in the gas phase and as monolayer on Au(111) surface are shown in Figure S1 of the Supporting Information. Table 1 summarizes the results reporting the position of the peaks in terms of their binding energy (BE), referred to the vacuum level and to the Fermi level for the gas phase and the monolayer respectively (i.e., the solid state BEs differ from the gas phase ones for the work function of the sample).^57^ Data are compared with the calculated ΔSCF values for the isolated molecule and for the H–B model (see Table 1), both of them referred to the vacuum level.

**Table 1:** Experimental and ΔSCF Calculated N 1s, O 1s, and C 1s BEs of Gaseous/Isolated CA, CA Monolayer on Au(111), and H–B Model^a^

experimental BEs (eV)		
	CA gas phase	CA/Au(111)
N (H-donor)	407.3	400.0
O (H-acceptor)	538.4	531.2
C	296.2	289.1

ΔSCF BEs (eV)		
	CA	H–B model
N (H-donor)	406.99	406.12
O (H-acceptor)	537.50	537.10
C	295.97	295.48

a Gas phase BEs are referred to the vacuum level, while those of CA monolayer on Au(111) are referred to the Au Fermi level (for a more direct comparison, monolayer BEs should be corrected for the work function sample).

The effect of the intermolecular bonding is evident considering the energy difference between the calculated BEs of the gas phase CA and the H–B model for each site considered in Table 1. The decrease of the BEs in going from CA to H–B model is more pronounced for the N site compared to the O site, in agreement with a donor–acceptor behavior of N/O sites of interacting molecules. This fingerprint of H-bonding is not evident in the experimental data. On the contrary, as we will discuss in the following, the N *K*-edge NEXAFS experimental spectra are in full agreement with the presence of H-bonding scheme. We suggest that the molecule–substrate interaction, not considered by calculations, plays a relevant role in the determination of the binding energies of the monolayer, dampening the effects due to the intermolecular bonding. Looking more in detail at the calculated values and considering the trend of the calculated BEs from CA to H–B model, a decrease of the BEs is obtained for all the three core-holes possibly due to a higher core-hole shielding effect promoted by the intermolecular H-bond interactions. The negative energy shift is larger for the N 1s core-hole (about 0.9 eV) than for the other two edges (less than 0.5 eV), confirming that the N atoms are acting as H-donor.^22^ The ΔSCF BEs of the dimer model are reported in the Table S1 of the Supporting Information and are useful to analyze in more detail the effects of the H-bond formation on the core levels of both interacting and noninteracting heteroatoms with respect to the monomer. In the dimer, the H-bond formation breaks the equivalence of the core–hole sites, as indicated by the labels of Figure 1. In the upper ring of the dimer (H-donor ring), the 1s level of the N15 (H-donor) site directly involved in the H-bond decreases by 0.84 eV compared to the monomer, in agreement with the trend observed for the H–B model, while the 1s level of N14 remains almost unperturbed (406,78 eV in the dimer and 406,99 eV in the monomer), since its chemical environment does not significantly change with respect to the monomer. In the bottom ring of the dimer (H-acceptor ring) the O6 O(H-acceptor) site is affected by the H-bond formation and its BE accordingly increases by 0.4 eV compared to CA while the BE of the O8 site is very similar both in the dimer and in the monomer (537.57 eV versus 537.50 eV). This analysis indicates that the H-bond formation affects more significantly the BE of the N (H-donor) site than the O (H-acceptor) site ^22,^.^58^ A final comment concerns a trend of the dimer BEs observed for all the core-site (see Table S1 of the Supporting Information): the BEs of the core sites of the (H-donor) ring are lower than those of the corresponding sites of the (H-acceptor) ring. This trend can be related to the H-bond interaction between the two rings which increases the charge density on the N15 site of the (H-donor) ring as well as on all the other core sites for delocalization effects, with respect to the (H-acceptor ring) core sites.

## NEXAFS Spectra

In the following discussion, the experimental N, O and C *K*-edges NEXAFS spectra are compared with the results obtained with the DFT-TP/B3LYP computational scheme. Only in the case of the N 1s core excitations has the theoretical description required the employment of computational approaches beyond the DFT-TP one, specifically TDDFT, in order to reach the best match with the experimental data.

**Figure 2:**
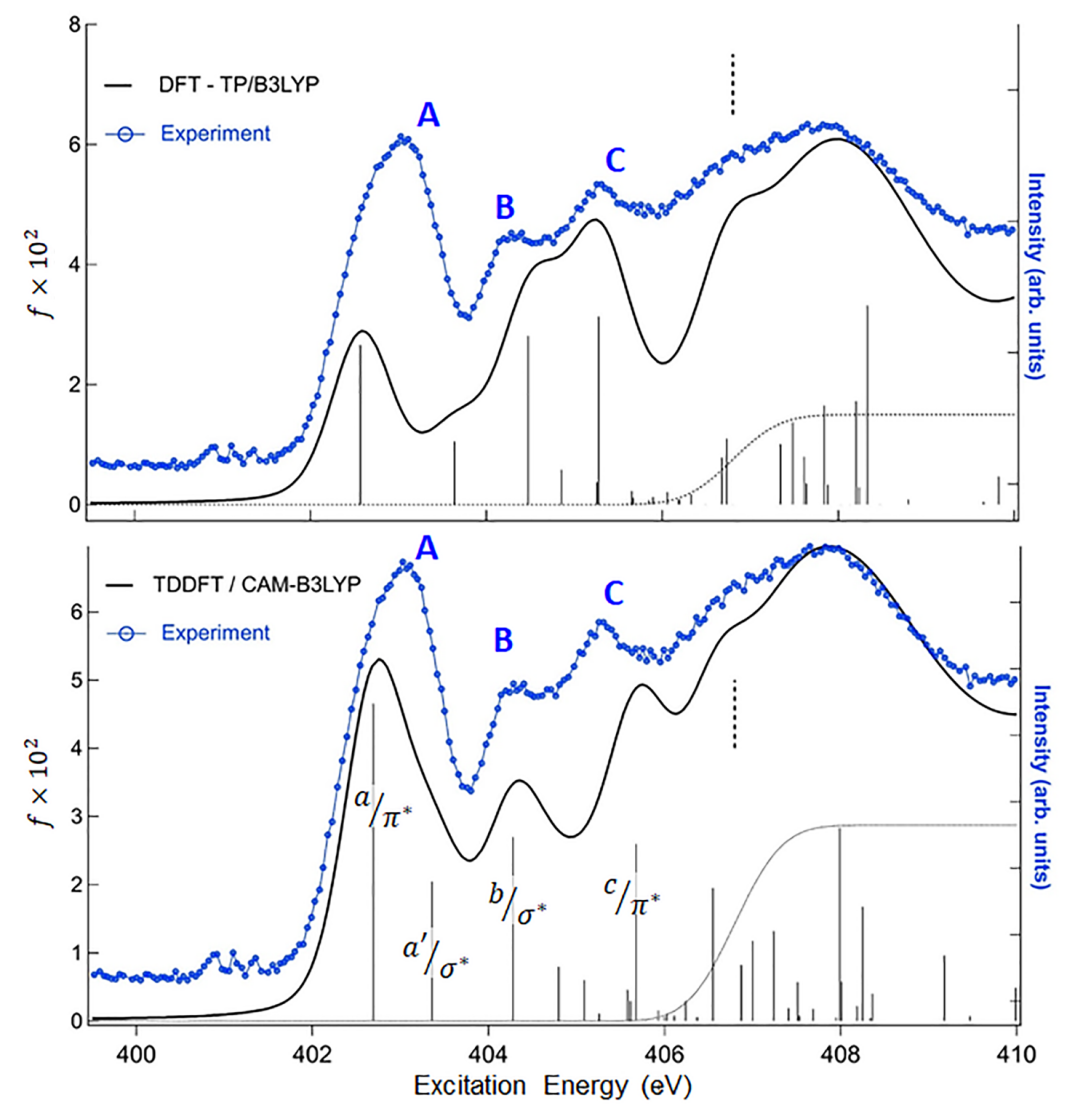
N *K*-edge gas phase NEXAFS spectrum of CA. Upper panel: comparison with the theoretical DFT-TP results shifted by −0.4 eV on the experimental energy scale. The ΔSCF theoretical N 1s IP is indicated with a vertical dashed line. Lower panel: comparison with the theoretical TDDFT/CAM-B3LYP results (shifted by +12.6 eV). The calculated N 1s IP (DFT-KS opposite eigenvalue) is indicated with a vertical dashed line. Experimental intensities are in arbitrary units and scaled in order to be compared with theoretical spectra.

We first consider the results for the N *K*-edge spectra of CA, which are expected to be the most affected by the H-bond formation in going from the gas-phase to the monolayer. The N 1s experimental and theoretical NEXAFS spectra of the gas-phase CA are presented in Figure 2. In particular, here we show the computed spectra obtained at DFT-TP and TDDFT/CAM-B3LYP levels, and the calculated excitation energies and oscillator strengths together with the assignment of the corresponding bands are available in the Supporting Information (Tables S2 and S3 of the Supporting Information).

Three main features (labeled A, B, and C) characterize the below edge region of the experimental spectrum followed by a large signal extending up above the ionization threshold. A low signal around 401 eV is also present due to residual N_2_ in the experimental chamber. The DFT-TP results (upper panel of Figure 2 and Table S2 of the Supporting Information) predict four main transitions associated with the A, B, and C features which correctly reproduce the energy separations among the experimental peaks while the intensity distribution is not satisfactorily described, in particular for the underestimate of the peak A intensity compared to peaks B and C. The use of the PW86x potential does not improve the DFT-TP results, so the disagreement with the experiment at DFT-TP level is not ascribable to the choice of the exchange–correlation potential. To investigate the possible origin of the discrepancies, we considered the possible effect of the coupling among the (1h1p) excitations by performing TDDFT calculations. The results obtained with the hybrid B3LYP xc kernel (TDDFT/B3LYP results), reported in Figure S3 and Table S3 of the Supporting Information, improve the intensity distribution indicating the influence of the configurations mixing. However, the discrepancy with the experiment appears minimized if the range-separated hybrid CAM-B3LYP is employed in the TDDFT calculations, as shown in Figure 2 (lower panel). As we can see in addition to a proper description of the intensity distribution, also the energy separation of the first two transitions is reduced allowing to assign with confidence the quite intense experimental peak A to two transitions, indicated as a/π* and a′/σ* in Figure 2. The TDDFT/CAM-B3LYP results are collected in Table S3 of the Supporting Information and compared with the TDDFT/B3LYP results. The analysis of the TDDFT eigenvectors highlights that CAM-B3LYP yields a stronger coupling among (1h1p) core excitations compared to B3LYP, furthermore it describes more appropriately the several σ* valence–Rydberg transitions that contribute to the N 1s spectrum. This is consistent with the capability of the range-separated functionals, such as CAM-B3LYP, to better describe the Rydberg excitations compared to the B3LYP functional.^59,60^ The present results therefore prove the effectiveness of the TDDFT/CAM-B3LYP computational scheme in describing the N 1s core excitations confirming what emerged in a previous theoretical investigation on the N 1s NEXAFS spectra of indole and its derivatives.^61^

It is important to point out that the nature of the main transitions contributing to the N 1s spectrum does not change in passing from the DFT-TP to the TDDFT approach, as can be deduced by a careful analysis of the results reported in Tables S2 and S3 of the Supporting Information. This is an important issue taking into account that the strong configuration mixing of TDDFT/CAM-B3LYP results, which provide the best description of the N 1s spectrum, makes more complex the assignment of the experimental features, and it is therefore useful to take advantage of the DFT-TP results which provide a key for the interpretation of the spectrum in terms of single particle description. In particular, the four most intense excited states of the TDDFT/CAM-B3LYP spectrum (indicated as a/π*, a′/σ*, b/σ*, and c/π* in Figure 2) can be correlated to the four main below edge transitions of the DFT-TP spectrum, allowing one to assign the A, B and C experimental peaks as explained in the following. Peak A is contributed by two excitations (a/π*, a′/σ*) which can be correlated to the N 1s→ 5b_1_ and N 1s→ 12a_1_ transitions of DFT-TP spectrum and have π*(C=O, C=N) and σ*(N–H) valence character, respectively. The main excited state contributing to peak B (b/σ*) has a mixed σ*(N–H)/Rydberg nature (N 1s→ 13a_1_ excitation in DFT-TP spectrum) while the peak C mainly derives from a mixed π* valence/Rydberg excitation (c/π*) (N 1s → 7b1 excitation in the DFT-TP spectrum). Representations of the aforementioned DFT-TP MOs are reported in Figure S2 of the Supporting Information. The higher energy transitions toward threshold involve final MOs, both of σ* and π*symmetry, with an increasingly diffuse Rydberg character.

**Figure 3:**
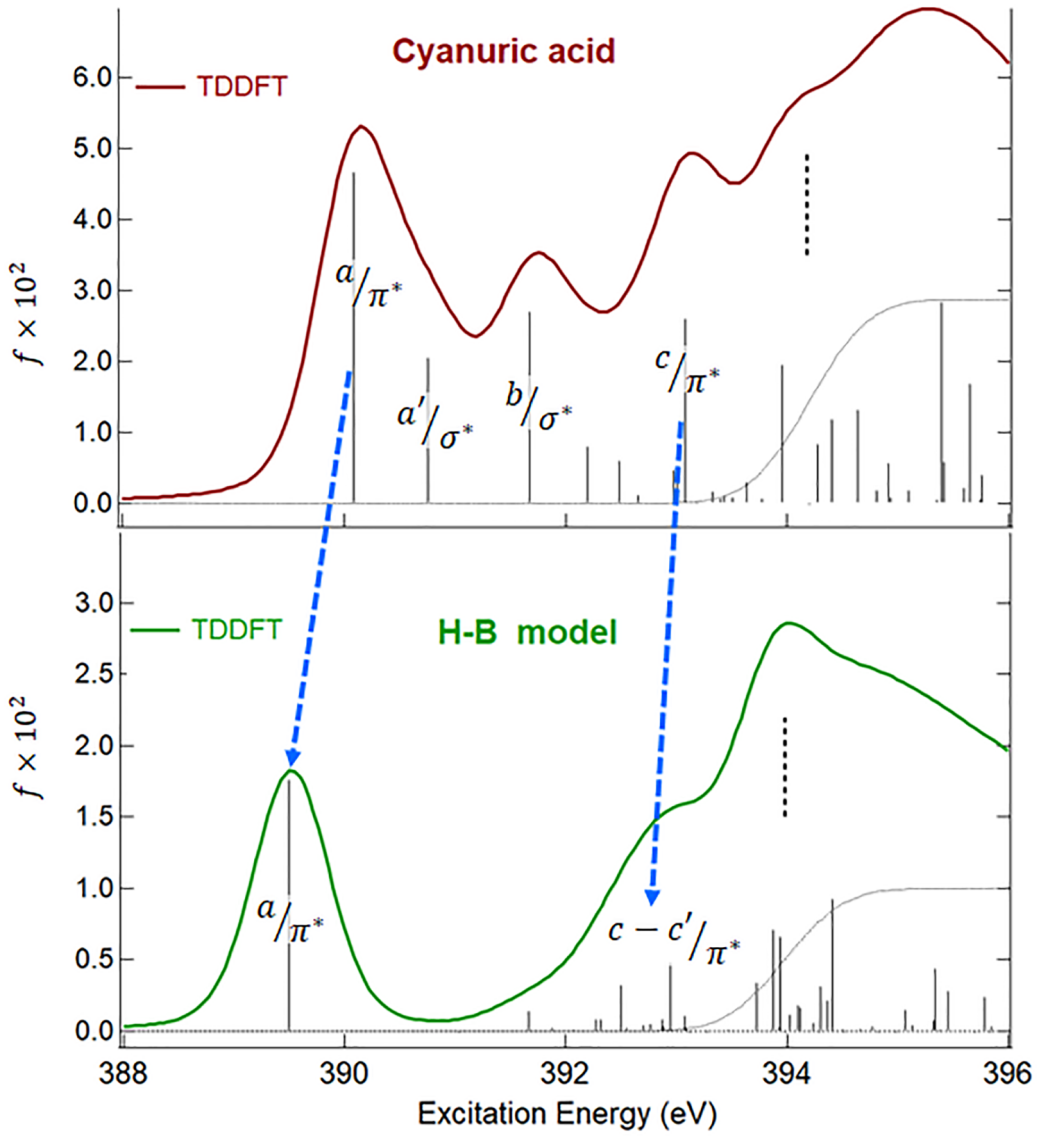
Theoretical TDDFT/CAM-B3LYP N 1s NEXAFS spectra of CA (upper panel) and the H–B model (lower panel). The calculated N 1s IPs (DFT-KS opposite eigenvalue) are indicated with a vertical black dashed line. The blue dashed lines are used to indicate related transitions in the two spectra.

The effect of the H-bond formation on the N 1s core excitations can be analyzed by comparing the theoretical N 1s NEXAFS spectra of CA and H–B model. We calculate the N 1s spectrum of the H–B model both at DFT-TP and TDDFT/CAM-B3LYP level since this system is used as a theoretical benchmark for the CA monolayer on Au(111) surface to compare the best theoretical spectrum with the experimental one in the solid state. Figure 3 reports the TDDFT/CAM-B3LYP N 1s spectrum of the H–B model and its comparison with the N 1s spectrum of the CA monomer while this comparison at the DFT-TP level is shown in Figure S4 of the Supporting Information. The list of the TDDFT/CAMB3LYP excitation energies and oscillator strengths of the H–B model are summarized in Table S4 of the Supporting Information.

A strong mixing of configurations affects the N 1s excited states of the H–B model, as in the case of TDDFT/CAM-B3LYP calculations of the CA monomer, therefore the DFT-TP calculated spectrum of the H–B model is still useful to support the attribution of the main spectral features. The H-bond formation significantly affects the N 1s spectrum of the H–B model compared to that of CA, and the most evident outcome is the reduced number of below edge resonances. Only two main peaks remain in the H–B model spectrum, which essentially correspond to the N 1s transitions of π* character. Considering in detail the TDDFT/CAM-B3LYP results of Figure 3, the lower energy peak is contributed by a single excitation at 389.50 eV, indicated as a/π*, while several excited states contribute to the second peak, the most intense ones at 392.50 and 392.95 eV indicated as c-c′/π*, respectively. A strong (1h1p) coupling affects the first most intense excitation, described by transitions to purely π* MOs, as well as the higher π* excited states which have a mixed π* valence/Rydberg character. The DFT-TP N 1s spectrum of the H–B model (Figure S4 of the Supporting Information) also exhibits only two main transitions (a/π* and c/π*) of π* nature (the final MOs are shown in Figure S4).

Figure 3 also highlights the correspondence among the peaks of π* nature in CA and H–B model spectra, which substantially maintain the same origin (main localization on the s-triazine ring and (C=O) ketonic bonds) and turn out to be unperturbed by the H-bond formation, also as concerns their energy separation (about 3.2 eV in H–B spectrum and 2.99 eV in CA spectrum). The two main transitions of σ* character of the CA spectrum (indicated as a′/σ* and b/σ* in the upper panel of Figure 3) are instead quenched in the H–B model spectrum. Since the two σ* transitions of the monomer involve MOs mainly localized on the N–H bonds, as previously discussed, their suppression in the H–B model probes that the H-bond formations of the central CA molecule with the O atoms of the surrounding CA molecules entail a significant rehybridization of the σ MOs in the energy range between the two main π* peaks. A similar quenching of the σ* transitions has been also observed in a previous study on the N *K*-edge spectra of the free melamine and involved in intermolecular H-bonds.^22^

To complete the analysis of the H-bonding effect on the N 1s core excitations we briefly report on the theoretical results for the dimer, which are collected in Table S5 and Figure S5 of the Supporting Information. Only the DFT-TP approach has been employed to calculate the N 1s spectrum of the dimer, since it can be useful only to discuss the evolution of the main features in going from the monomer to the H–B model. To this purpose, Figure S5 of the Supporting Information compares the N 1s spectrum of the dimer with those calculated at DFT-TP level for the CA and the H–B model. The shape of the dimer spectrum does not show significant differences with respect to the CA spectrum and four main peaks (labeled 1, 2, 3, and 4 in Figure S5) are still present with very similar intensity distribution as in the monomer. It is complex to identify a clear correspondence between the features of the dimer and the monomer, since the equivalence of the N atoms is broken in the dimer and the nonequivalent N sites give different contributions to the total N 1s spectrum. These contributions are highlighted in Figure S5 of the Supporting Information and their analysis can provide some interesting insights, in particular if we look at the N14 and N15 sites of the dimer donor ring that give rise to partial profiles quite different. The N15 profile is shifted to lower energy with respect to N14, in agreement with the trend followed by the relative IPs; furthermore, only two peaks below the edge are present compared to the four peaks of the N14 profile. The two N15 peaks have π* nature as the first and fourth peaks of N14 while the intermediate peaks of N14 have mainly σ*(N–H) characters. The profiles of the N1 and N11 sites are similar to that of N14. In summary, only the spectrum of the N15 site is affected by the H-bond formation and shows a profile similar to that of the H–B model while to the other N sites of the dimer give a spectral response similar to that of the monomer. By summing up all the profiles the contribution of N15 is smeared therefore the total profile is conditioned by the contributions of the other N sites and resembles the profile of the monomer.

**Figure 4:**
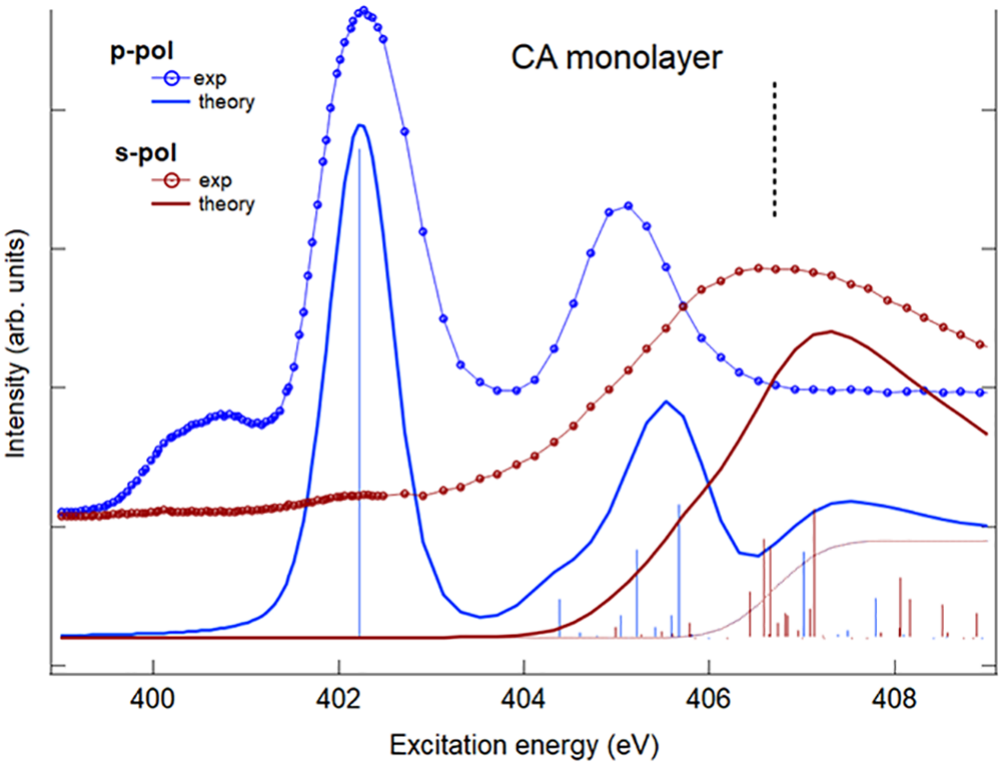
N *K*-edge NEXAFS spectra of cyanuric acid on Au(111) surface at perpendicular (p-pol) and parallel (s-pol) polarization of the light with respect to the surface. The TDDFT/CAM-B3LYP calculated spectra of the fixed in space H–B model have been shifted by 12.72 eV on the experimental energy scale. N 1s IP (DFT-KS opposite eigenvalue) is indicated with a vertical black dashed line.

The N 1s results discussed so far prove that this core level is a valuable probe of the H-bond interaction and can be therefore useful to explore the self-assembling of the CA molecules on the Au(111) surface. To this purpose, Figure 4 compares the N 1s NEXAFS experimental polarized spectra of CA adsorbed on the Au(111) surface with the TDDFT/CAM-B3LYP theoretical ones for the H–B model. The experimental spectra have been acquired with two light polarizations: parallel to the surface, (*s-pol* spectrum), and perpendicular to the surface (*p-pol* spectrum). The p-pol and s-pol experimental spectra of the CA monolayer in Figure 4 show a large dichroism between the two polarizations. In particular, π* resonances have maximum intensity in p-pol, traced to a flat adsorption geometry of the CA on the Au surface. To compare the theoretical results with the experiment we have calculated the p- and s-pol spectra of the gas-phase H–B model system oriented with the molecular plane fixed in the *xy* plane of the molecular frame. This choice allows one to check if the H–B model is able to capture the essential nature of the 2D framework and of the H-bond intermolecular interaction, leaving to future investigations the modeling of the system with the underlying Au(111) surface which is needed to analyze the role of the molecule–substrate interaction. The agreement between experiment and theory is satisfactory, although the feature present in the p-pol experimental spectrum at lower energy (around 401 eV) is not described by the calculation. We believe that such structure could derive from a possible interaction of the NH group with the surface, as suggested by previous studies evidencing the amine–Au(111) affinity and its influence on N 1s NEXAFS spectra.^62−64^

The calculated p-pol component is prevalent in the below edge region and correctly reproduces the remarkable dichroic effects observed in the experiment. The two below edge peaks of the p-pol spectrum (around 402 and 405.5 eV) derive from the transitions of π* symmetry that were labeled as a/π* and c-c′/π* in the total H–B model spectrum (see Figure 3) previously described. In the region of the calculated second p-pol peak (around 405.5 eV) the s-components are present only with negligible contributions while they acquire intensity at higher energy just around the ionization threshold where transitions toward mainly diffuse states of σ symmetry are dominant. Around 407 eV also the p polarization component gives less intense contributions associated with out of plane transitions of diffuse nature so that the intensity trend of the p-pol experimental component is correctly described by the calculation. The spectral shape of the s-pol component confirms the quenching of the σ* resonances localized on the N–H groups in the presence of H-bond intermolecular interaction among the CA molecules The good agreement found between experiment and theory applied to the oriented molecule proves the ability of the H–B model to capture the main effects of the H-bond intermolecular interactions between the CA molecules in the monolayer furthermore proves that the Au surface does not perturb significantly the N 1s transitions in the energy region above 402 eV. Further studies are needed to include the surface in the model and to confirm that the surface–monolayer interaction account for the p-pol experimental signal below 402 eV.

**Figure 5:**
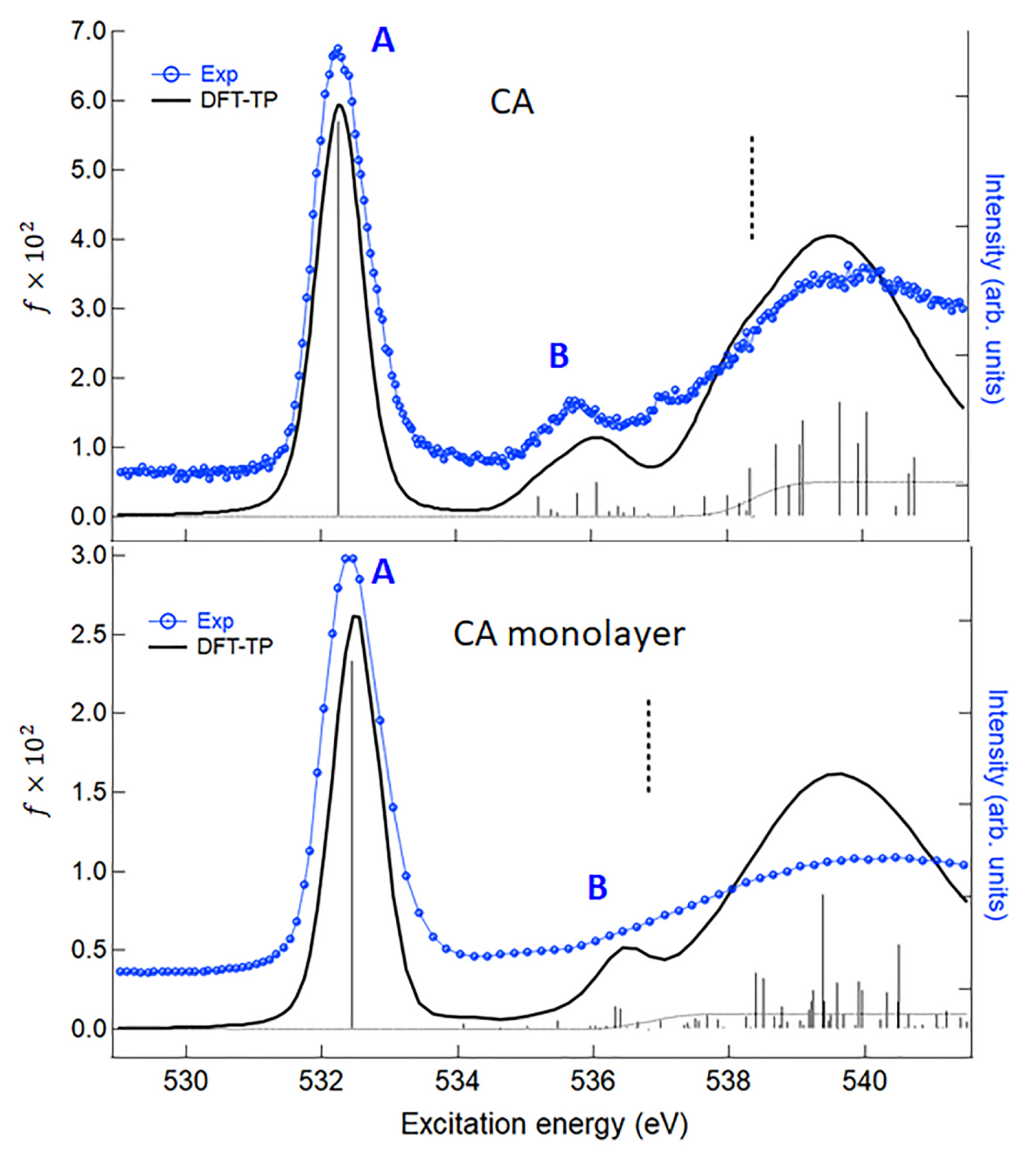
O *K*-edge NEXAFS spectrum of CA in gas-phase and adsorbed on Au(111). Upper panel: comparison between the experimental gas-phase spectrum with the theoretical DFT-TP results shifted by −0.57 eV on the experimental energy scale. The calculated ΔSCF O 1s IP is indicated with a vertical dashed line. Lower panel: comparison between the experimental unpolarized spectrum of CA monolayer and the theoretical DFT-TP spectrum of the H–B model. The experimental curve is obtained as a p-pol + 2(s-pol) sum. The theoretical results for H–B model are shifted by −0.29 eV on the experimental energy scale. The calculated ΔSCF O 1s IP is indicated with a vertical dashed line. Experimental intensities are in arbitrary units and scaled in order to be compared with the theoretical spectra.

Consider now the O *K*-edge NEXAFS results. The O 1s experimental spectrum of gas phase CA is reported and compared with the theoretical DFT-TP spectrum in Figure 5. In order to discuss the effect of the H-bond interaction on the spectral features, Figure 5 also compares the unpolarized experimental spectrum of the CA monolayer with the calculated DFT-TP spectrum of the H–B model. The experimental spectrum is obtained as the sum (p-pol + 2(s-pol)), which holds in the case of a three-fold or many-fold substrate.^65^ The list of the DFT-TP calculated excitation energies and oscillator strengths together with the assignment of the spectral features of CA and H–B model are reported in Tables S6 and S7 of the Supporting Information.

The experimental O 1s absorption spectrum of CA is dominated by a strong symmetric peak (labeled A) followed by a broad and less intense resonance (labeled B) around 535.6 eV. A further intense resonance appears just above threshold. The DFT-TP calculated spectrum describes very satisfactorily the experiment both as concerns the energy separation and the intensity distribution among the spectral features. Peak A is assigned to the O 1s transition to the LUMO orbital of π* character, mainly localized on the C=O bond of the O site with the core hole. Several low intensity transitions of both valence and Rydberg characters contribute to the spectral region of peak B; the most intense one is relative to the 7b_1_ π* final orbital (see Table S6 of the Supporting Information) with the others toward σ*(N–H)/Rydberg final MOs (the 3D plots of some final DFT-TP MOs are reported in Figure S6 of the Supporting Information). The nature of the O 1s transitions contributing to the feature B reflects that of the N 1s transitions in the region of B and C peaks; however, the intensity is strongly reduced for the reduced participation of O *n*p atomic components to the final MOs, and all the transitions are clustered in the single B resonance.

The presence of intermolecular H-bonds induces small differences in the shape of the O 1s photoabsorption spectrum of the CA monolayer: in the experiment, structure B is embedded in a smooth signal, and in the H–B model, the spectrum slightly changes its shape due to the intensity quenching of the σ* transitions. The only transitions with not negligible intensity contributing to B feature have in fact π* nature, as shown in Table S7 of the Supporting Information. Since all the calculated transitions contributing to peak B both in the CA and in the H–B model spectra have low intensity, their relevance to probe the H-bond effects is negligible and is not appreciable at experimental level.

The O 1s NEXAFS spectrum of the dimer does not add significant information, and the spectral variations of the dimer with respect to the CA spectrum are negligible, as shown in Figure S7 of the Supporting Information and there briefly commented upon.

**Figure 6:**
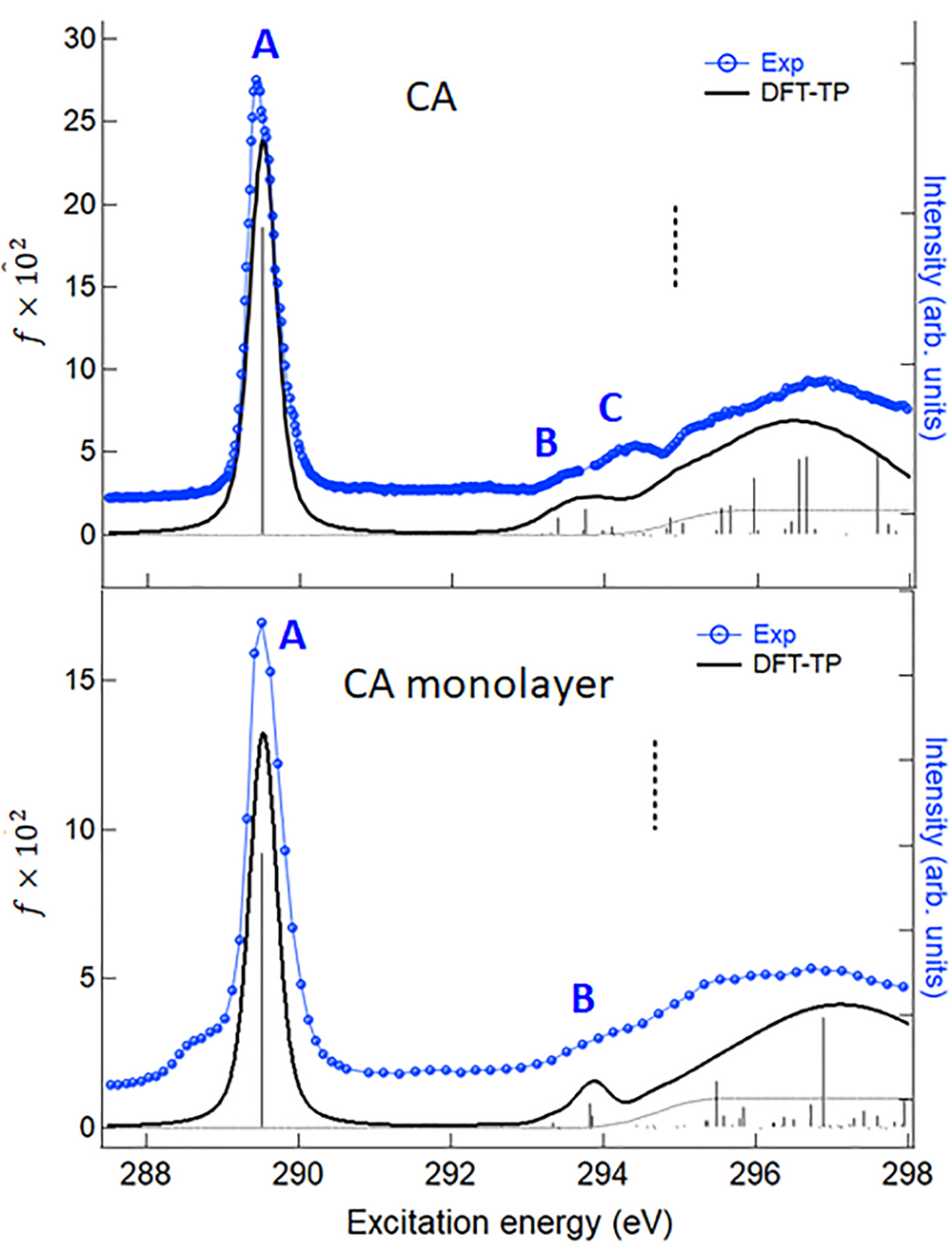
C *K*-edge gas phase NEXAFS spectrum of CA in gas-phase and adsorbed on Au(111). Upper panel: comparison between the experimental gas-phase spectrum with the theoretical DFT-TP results shifted by −1.05 eV on the experimental energy scale. The calculated ΔSCF C 1s IP is indicated with a vertical dashed line. Lower panel: comparison between the experimental unpolarized spectrum of CA monolayer and the theoretical DFT-TP spectrum of the H–B model. The experimental curve is obtained as p-pol + 2(s-pol) sum. The theoretical results for H–B model are shifted by −0.80 eV on the experimental energy scale. The calculated ΔSCF C 1s IP is indicated with a vertical dashed line. Experimental intensities are in arbitrary units and scaled in order to be compared with the theoretical spectra.

We finally present the C *K*-edge NEXAFS results. Figure 6 reports the experimental C 1s spectrum of gas-phase CA compared with the DFT-TP theoretical spectrum as well as the experimental unpolarized C 1s spectrum (p-pol +2(s-pol)) of the CA monolayer compared with the DFT-TP theoretical spectrum of H–B model, to point out possible effects of the H-bond interaction. The main DFT-TP excitation energies and oscillator strengths of both systems are reported in Tables S8 and S9 of the Supporting Information.

An intense resonance dominates the spectrum (peak A) well separated in energy from two less intense and broad features (B and C) then a further large resonance appears just around the edge. The DFT-TP calculations assign the first peak A to the C 1s → LUMO (π*) transition and associate the structure B to several low intensity transitions of mainly π* nature. The stronger intensity of the LUMO transition compared to the higher energy π* ones can be related to the greater localization of this final MO on the C=O bond involving the C core-hole site. Feature C is contributed by many low intensity excitations toward final MOs of mainly σ*(N–H) character mixed with diffuse components. (The plots of some final DFT-TP MOs are reported in Figure S8 of the Supporting Information.)

The higher energy part of the spectrum toward the threshold involve transitions of mainly diffuse nature. The experimental unpolarized spectrum of the CA monolayer is quite similar to the CA spectrum apart from the lower energy shoulder of the peak A which can be attributed to a molecule–substrate interaction, similarly to what discussed for the N 1s spectrum. The calculated feature C (around 295 eV), is missing in the H–B model spectrum while the nature and relative position of both the A and B features is very similar to the CA spectrum, therefore confirming the origin of the spectral change following the H-bond interaction, already discussed for the N and O *K*-edges. Since the differences between the C 1s NEXAFS spectra of CA and H–B model concern spectral features of very low intensity, they are not particularly relevant and in fact are not evident experimentally, as in the case of the O 1s spectra.

The analysis of the DFT-TP results for the dimer (reported in Figure S9 of the Supporting Information) and the comparison with the CA and H–B model results confirm the small effect that the H-bond interaction has on the C 1s core spectra. Only the quenching of the C feature present in the CA spectrum can be observed, which is also absent in the H–B model spectrum.

## Conclusions

The main goal of the present study is to understand how intermolecular H-bonding interactions affect the local electronic states of the cyanuric acid by means of core level XPS and NEXAFS spectroscopic investigations. To this purpose, we have first measured and interpreted, by means of DFT calculations, the core spectra at the N 1s, O 1s and C 1s ionization thresholds of the gas-phase CA. It has been shown that the DFT-TP scheme is accurate enough to describe the O 1s and C 1s NEXAFS spectra of CA while the TDDFT approach is needed, in conjunction with the range-separated XC CAM-B3LYP functional, to obtains a correct reproduction of the N 1s experimental spectrum. The effect on the core spectra of the H-bond interaction has been then investigated by considering the CA monolayer adsorbed on Au(111) surface, modeled at theoretical level by a periodic arrangement of the CA monomers thorough H-bond in a hexagonal lattice (H–B model). The H-bond formation modifies the local electronic state of the N–H donor lowering the BE of the N 1s level while the O 1s level is less affected by accepting a proton. The analysis of the excitations from all the three N, C, and O sites indicates that the σ* empty states localized on the N–H group are the mostly affected by the H-bond intermolecular interaction while the π* empty states, mainly localized on the ring and the C=O bonds, remain substantially unchanged. The σ*(N–H) features have significant intensity only in the N 1s core spectrum of the CA monomer and their quenching in the CA monolayer spectrum is a significant probe of the H-bond formation. This characterization has been further validated by the analysis of the N 1s polarized spectra of the CA monolayer on the Au(111) surface and constitutes an essential step for future investigation aimed at understanding the H-bond interaction between CA and melamine to build-up highly ordered 2D networks on the surface.

## Supporting Information Available

Experimental XPS spectra of CA in gas-phase and adsorbed on Au(111) (Figure S1); calculated BEs for the CA dimer (Table S1); list of calculated excitation energies, oscillator strengths, and peaks assignment for the N 1s NEXAFS spectra of CA gas phase (Tables S2 and S3), the H–B model (Table S4), and the CA dimer (Table S5); O 1s NEXAFS spectra of CA gas phase (Table S6) and H–B model (Table S7); C 1s NEXAFS spectra of the CA gas phase (Table S8) and H–B model (Table S9); comparison between TDDFT/B3LYP and TDDFT/CAM-B3LYP N 1s NEXAFS spectra (Figure S3 and Table S3); comparison between DFT-TP N 1s NEXAFS spectra of the CA gas-phase and the H–B model (Figure S4); comparison among the DFT-TP NEXAFS spectra of CA gas-phase, CA dimer, and H–B model for N *K*-edge (Figure S5), O *K*-edge (Figure S7), and C *K*-edge (Figure S9); and 3D plots of selected molecular orbitals of the systems (Figures S2, S6, and S8) (PDF)
