# Supplementary material for: Probing Intermolecular H-Bonding Interactions in Cyanuric Acid Networks: Quenching of the N K-Edge Sigma Resonances
Source: J Phys Chem A. 2022 Sep 28;126(39):6870–81. doi: 10.1021/acs.jpca.2c04517 (PMC9549465; doi:10.1021/acs.jpca.2c04517)
Supplement: Supplementary file 1 — jp2c04517_si_001.pdf [file jp2c04517_si_001.pdf]

Supporting Information

Probing Intermolecular H-bonding Interactions in Cyanuric Acid Networks: Quenching of the N K-edge Sigma Resonances

Valeria Lanzilotto[a,c], *Daniele Toffoli*[b,c], *Elisa Bernes*[b], *Mauro Stener*[b,c], *Elisa Viola*[a], *Albano Cossaro*[b,c] , *Roberto Costantini*[c] , *Cesare Grazioli*[c], *Roberta Totani*[d], *Giovanna Fronzoni*[b]*

[a] Department of Chemistry, Sapienza Università di Roma, P.le A. Moro 5, Roma, 00185, Italy

[b] Department of Chemical and Pharmaceutical Sciences, University of Trieste, 34127 Trieste, Italy.

[c] IOM-CNR, Istituto Officina dei Materiali-CNR, S.S.14, Km 163.5, 34149 Trieste, Italy.

[d] ISM-CNR, Istituto Struttura della Materia-CNR, LD2 Unit, S.S. 14, Km 163.5, 34149 Trieste, Italy

*Corresponding author: fronzoni@units.it

1. Experimental and theoretical XPS results

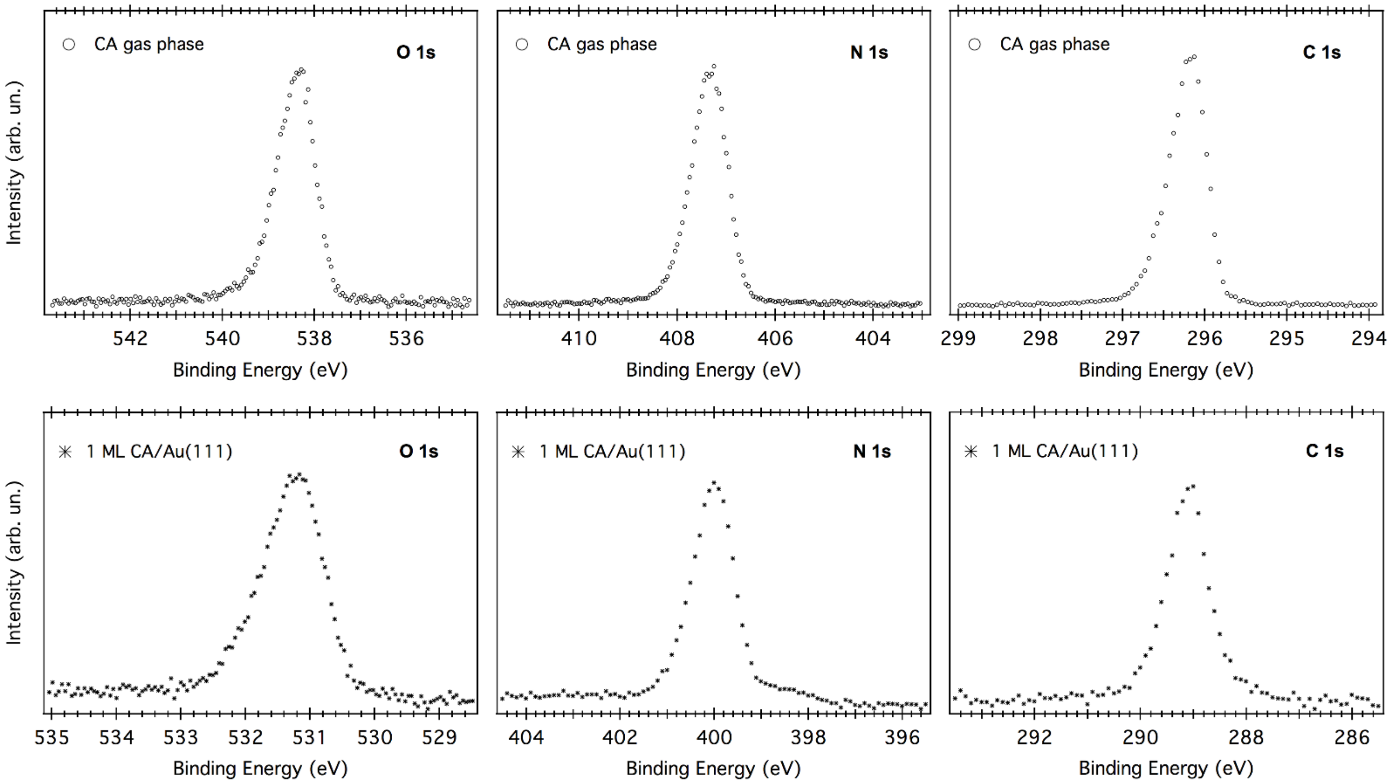

Figure S1. Experimental O1s, N1s and C1s XPS spectra of CA. Upper panel: gas-phase CA; lower panel: CA monolayer on Au (111).

Table S1. Calculated ΔSCF N1s, O1s and C1s binding energies for the CA dimer

Site[a]	BE (eV)
N14 (H-donor ring)	406.78
N15 (H-donor ring)	406.15
N1 (H-acceptor ring)	407.51
N11 (H-acceptor ring)	407.44
O19 (H-donor ring)	536.85
O20 (H-donor ring)	537.01
O6 (H-acceptor ring)	537.90
O8 (H-acceptor ring)	537.57
C13 (H-donor ring)	295.40
C16 (H-donor ring)	295.59
C3 (H-acceptor ring)	296.42
C7 (H-acceptor ring)	296.21

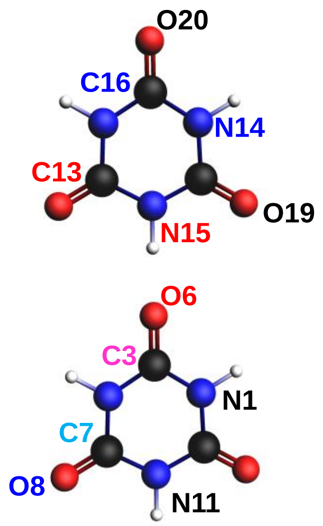

[a]The atom labels refers to the structure of the dimer reported on the right side. The (H-donor) ring corresponds to the upper ring of the dimer structure, the (H-acceptor) ring to the bottom ring.

2. Details on the NEXAFS spectra calculations

Table S2. Peak assignment of the DFT-TP N1s NEXAFS spectrum of gas phase CA: excitation energies (eV) and oscillator strengths *f* of the main below edge transitions.

Peak	E(eV)	[a] E(eV)	fx10^2	Assignment, main character of the final MO
A	402.96	402.56	2.66	5 b_1 / π*(C=O) + π*(C-N)
	404.04	403.64	1.05	12 a_1/ mixed σ*(N-H)/Rydberg
B	404.87	404.47	2.81	13 a_1/ mixed σ*(N-H)/Rydberg
	405.25	404.85	0.58	9 b_2/ mixed σ*(N-H)/Rydberg
C	405.66	405.26	0.37	14 a_1/ mixed σ*(N-H)/Rydberg
	405.67	405.27	3.13	7 b_1/π*(C=O) + π*(C-N)
	406.05	405.65	0.23	15 a_1/ Rydberg (in plane components)
Higher transitions	406.46	406.06	0.21	17 a_1/ Rydberg (in plane components)
	407.07	406.67	0.78	12 b_2 / mixed σ*(N-H)/Rydberg
	407.13	406.73	1.10	20 a_1 / mixed σ*(N-H)/Rydberg

[a] Calculated excitation energies shifted by -0.40 eV to match the first experimental peak.

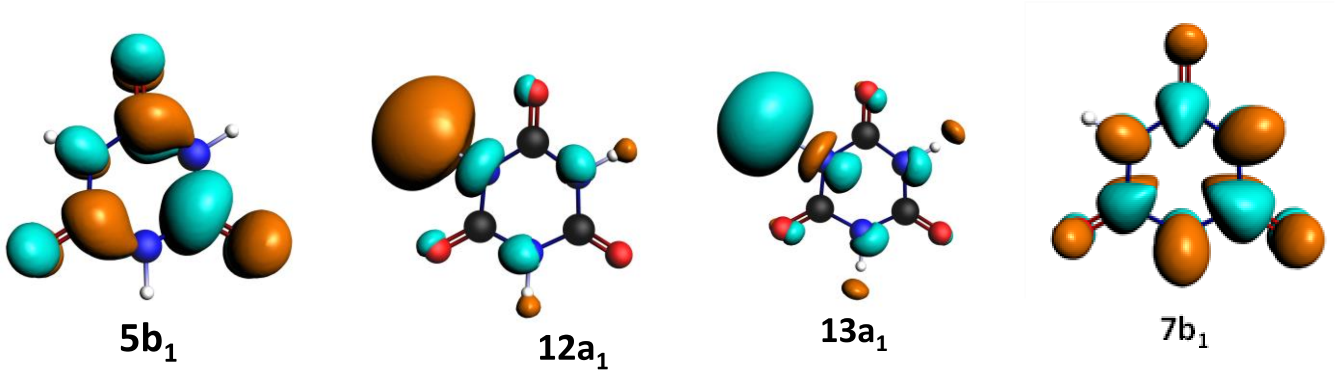

Figure S2. 3D plots of selected final MOs of CA relative to the N1s DFT-TP core excitation calculations. Displayed isosurface corresponds to ±0.03 e $^{1/2}$ $a_0^{-3/2}$ value for $5b_1$ and $7b_1$ MOs and to ±0.025 e $^{1/2}$ $a_0^{-3/2}$ value for $12a_1$ and $13a_1$ MOs.

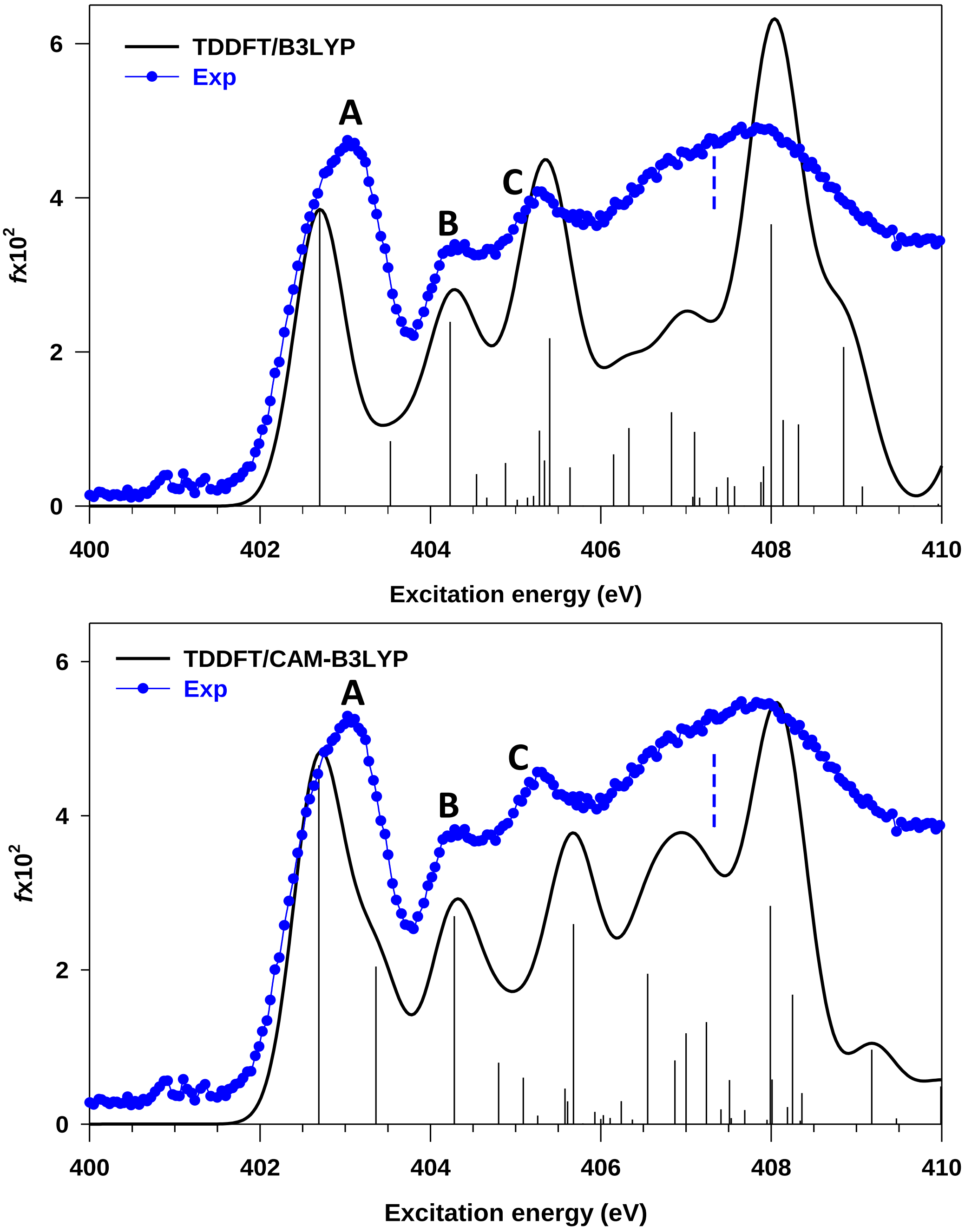

Figure S3. Comparison between the N K-edge gas phase NEXAFS spectrum of CA and theoretical TDDFT results. Upper panel: TDDFT/B3LYP spectrum shifted by +13.2 eV on the experimental energy scale. Lower panel: TDDFT/CAM-B3LYP spectrum shifted by +12.6 eV on the experimental energy scale. The stick spectra are broadened by using a Gaussian line shape with FWHM = 0.7 eV. The experimental N1s IP is indicated with a vertical blue dashed line.

Table S3. TDDTF/B3LYP and TDDTF/CAM-B3LYP excitation energies (eV) and oscillator strengths (fx10^2) of N1s NEXAFS spectrum of gas phase CA.

TDDFT/CAM-B3LYP				TDDFT/B3LYP		
Peak	E[a]	[b]fx10^2	[c]Major MO→MO transitions for each excitation	E[d]	[b]fx10^2	[c]Major MO→MO transitions for each excitation
A	402.69	4.66	N $1s^{-1}$- π*(C=O, C=N) ($6b_1$)/83%	402.70	3.83	N $1s^{-1}$ -π*(C=O, C=N) ($5b_1$)/96%
	403.36	2.05	N $1s^{-1}$-σ*(NH)/Rydberg($12a_1$)/44%<br>-σ*(NH)/Rydberg ($18a_1$)/22%	403.53	0.84	N $1s^{-1}$ σ*(N-H)/Rydberg($12a_1$) /87%
B	404.28	2.70	N $1s^{-1}$-σ*(NH)/Rydberg ($15a_1$)/21%<br>-σ*(NH) ($12a_1$) /18%	404.23	2.39	N $1s^{-1}$-σ*(N-H)/Rydberg ($13a_1$)/65%
C	405.68	2.59	N $1s^{-1}$- π*(C=N)/Rydberg ($9b_1$)/47%<br>- π*Rydberg ($10b_1$)/23%	405.40	2.18	N $1s^{-1}$-π*(C=O, C=N)/Ryd ($9b_1$)/70%<br>-π*(C=O)/ Rydberg ($7b_1$)/20%
Higher excitations	406.55	1.95	N $1s^{-1}$ - σ* Rydberg ($12b_2$)/42%<br>- σ* ($10b_2$) /19%	406.33	1.01	N $1s^{-1}$ - σ*Rydberg ($20a_1$) /87%
	407.00	1.18	N $1s^{-1}$ -π*Rydberg ($10b_1$)/46%<br>-π*(C=N)/Rydberg ($9b_1$) /22%	406.83	1.22	N $1s^{-1}$ - π*Rydberg ($10b_1$)/91%
	407.24	1.32	N $1s^{-1}$ - σ* Rydberg ($14b_2$)/60%	407.10	0.96	N $1s^{-1}$ - σ* Rydberg($13b_2$)/51%<br>- σ* Rydberg (14)b_2)/42%

[a] Calculated excitation energies shifted by +12.6 eV to match the first experimental peak.

[b] Only the main transitions contributing to the below edge peaks are reported.

[c] The numbering of the final MOs (given in brackets) of A_1 and B_2 symmetries has been adapted to the frozen core numbering of the DFT-TP calculations.

[d] Calculated excitation energies shifted by +13.2 eV to match the first experimental peak.

Table S4. TDDTF/CAM-B3LYP excitation energies (eV) and oscillator strengths (*f*x10^2) of the N1s NEXAFS spectrum of H-B model

Symmetry final state	E	[a]fx10^2	Major MO→MO transitions for each excitation
B_1	389.50	1.76	N $1s^{-1}$ - π*(C=O, C=N) ($26b_1$)/37%<br>- π*(C=O, C=N) ($28b_1$)/35%
B_1	392.50	0.32	N $1s^{-1}$ - π*(C=O, C=N) ($26b_1$)/31%<br>- π*(/Rydberg ($30b_1$)/20%
B_1	392.95	0.48	N $1s^{-1}$ - π*/Rydberg ($35b_1$)/20%<br>- π*/Rydberg ($31b_1$)/16%
A_1	393.72	0.34	N $1s^{-1}$ - σ*/ Rydberg ($109a_1$)/60
A_1	393.87	0.71	N $1s^{-1}$ - σ* Rydberg ($110a_1$)/22%<br>- σ* Rydberg ($109a_1$)/14%

[a] Only the main transitions contributing to the below edge peaks are reported.

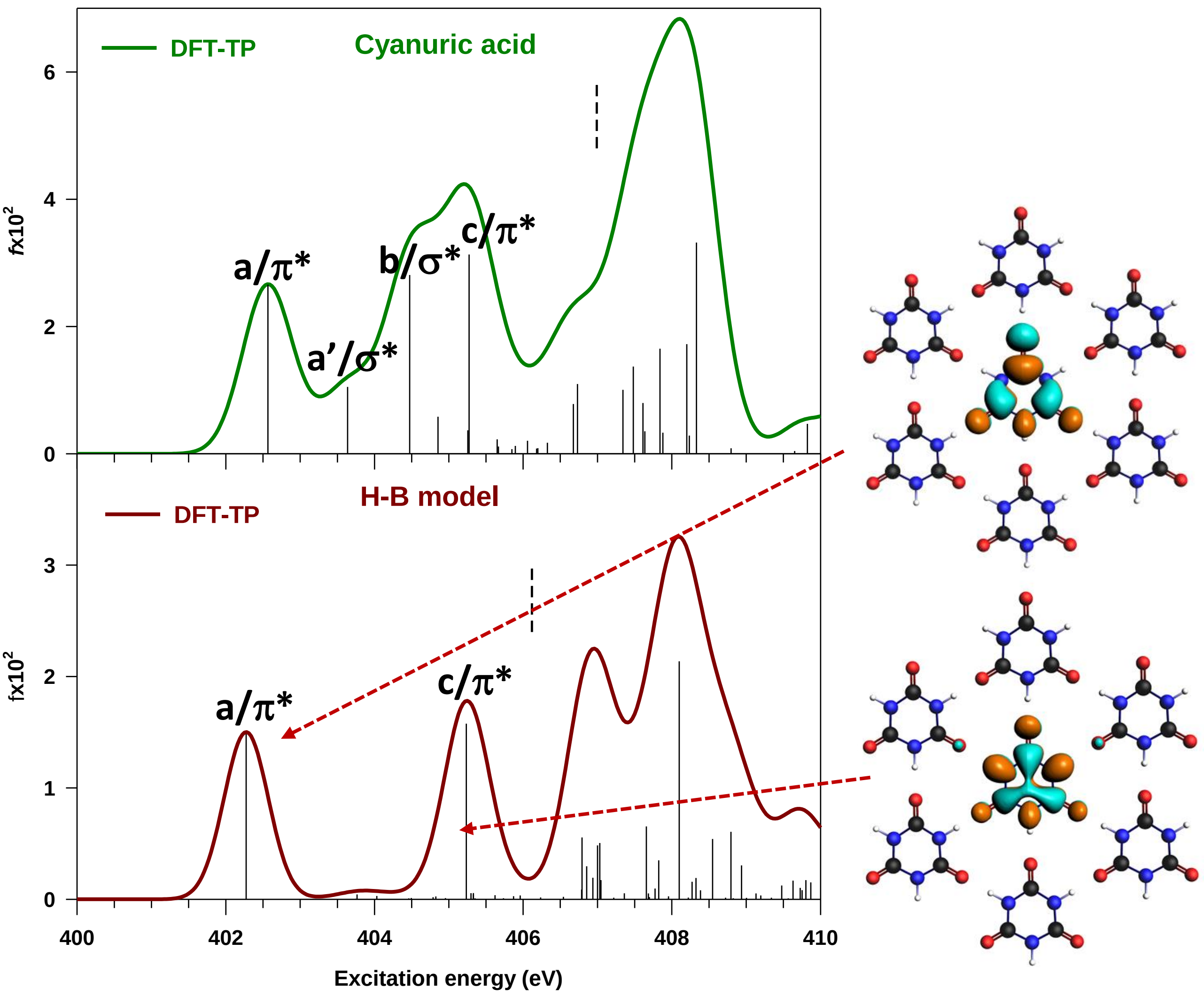

Figure S4. DFT-TP calculated N1s NEXAFS spectra of CA (upper panel) and H-B model (lower panel). The stick spectra are broadened by using a Gaussian line shape with FWHM = 0.7 eV. The ΔSCF calculated N1s IPs are indicated with a vertical dashed line. The 3D plots of the DFT-TP final MOs of H-B model relative to the two main transitions below edge are shown on the right side: displayed isosurface corresponds to ±0.03 $e^{1/2}$ $a_0^{-3/2}$ value.

Table S5. Peak assignment of the N1s NEXAFS spectrum of CA dimer: DFT-TP excitation energies (eV) and oscillator strengths *f* of the main below edge transitions.

Peak	Core-hole site	E(eV)	fx10^2	Assignment, main character of the final MO
**1**	N_{15}	402.55	0.85	14 A'' (LUMO+1) / π*(C=O) + π*(C-N)
	N_1	402.90	0.55	13 A'' (LUMO)/ π*(C=O) + π*(C-N)
	N_{11}	402.94	0.84	13 A''/ π*(C=O) + π*(C-N)
	N_{14}	403.15	1.53	14 A''/ π*(C=O) + π*(C-N)
	N_1	403.19	1.39	14 A''/ π*(C=O) + π*(C-N)
**2**	N_{14}	403.95	1.38	38 A'/σ*(N-H)
	N_{11}	404.13	0.59	38 A'/σ*(N-H)
	N_1	404.26	1.02	38 A'/σ*(N-H)
**3**	N_{14}	404.78	1.01	39 A'/σ*(N-H)/Rydberg
	N_{11}	405.07	0.88	39 A'/ σ*(N-H)/Rydberg
	N_1	405.17	1.98	39 A'/σ*(N-H)/Rydberg
	N_{15}	405.24	1.09	18 A''/ π*(C=O) + π*(C-N)
**4**	N_1	405.61	0.53	40 A'/ σ*(N-H)/Rydberg
	N_1	405.83	1.00	17 A''/ π*(C=O) + π*(C-N)
	N_{14}	405.89	2.04	18 A''/ π*(C=O) + π*(C-N)
	N_1	405.96	1.07	18 A''/ π*(C=O) + π*(C-N) / Rydberg

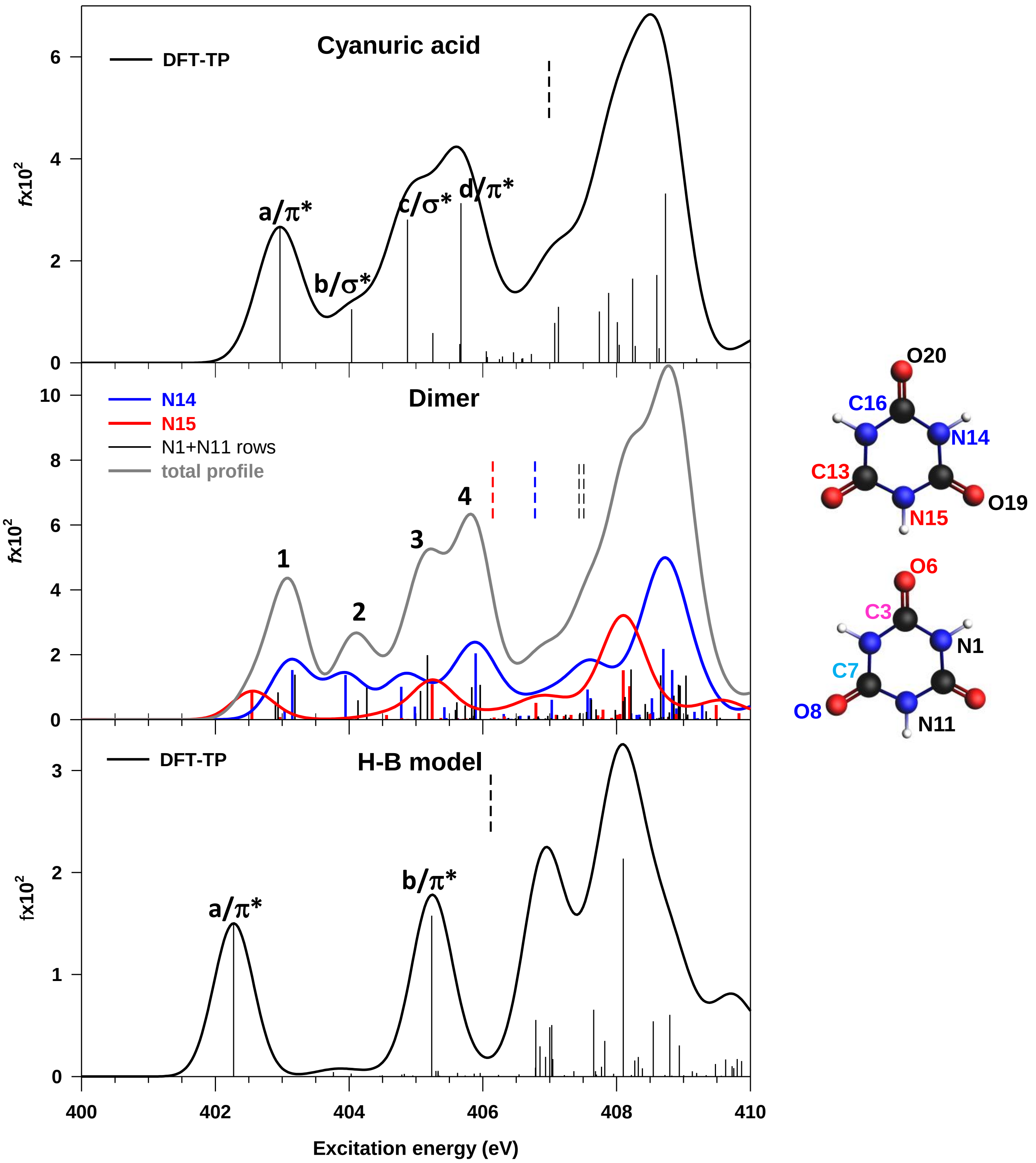

Figure S5. DFT-TP calculated N K-edge NEXAFS spectra of CA (upper panel), CA dimer (middle panel) and H-B model (lower panel). The partial contributions of the non-equivalent N atoms to the total spectrum of the dimer are also highlighted with different colours. The stick spectra are broadened by using a Gaussian line shape with FWHM = 0.7 eV. The calculated ΔSCF N1s IPs are indicated with vertical dashed lines. The chemical structure of the dimer is reported on the right side.

Table S6. Peak assignment of the O1s NEXAFS spectrum of gas phase CA: DFT-TP excitation energies (eV) and oscillator strengths *f* of the main below edge transitions.

Peak	E(eV)	[a]E(eV)	[b]fx10^2	Assignment, main character of the final MO
**A**	532.83	532.26	5.70	5 b_1/π*(C=O)+π*(C-N)
**B**	535.78	535.21	0.29	9 b_2/σ*(N-H)-Rydberg
	535.97	535.40	0.10	13 a_1/Rydberg
	536.36	535.79	0.34	14 a_1/σ*(N-H)-Rydberg
	536.64	536.07	0.50	7 b_1/π*(C=O)+π*(C-N)
	536.95	536.38	0.16	8 b_1/Rydberg
	537.19	536.62	0.14	18 a_1/Rydberg

[a] Calculated excitation energies shifted by -0.57 eV to match the first experimental peak.
[b] Only transitions with fx10^2≥0.10 are reported.

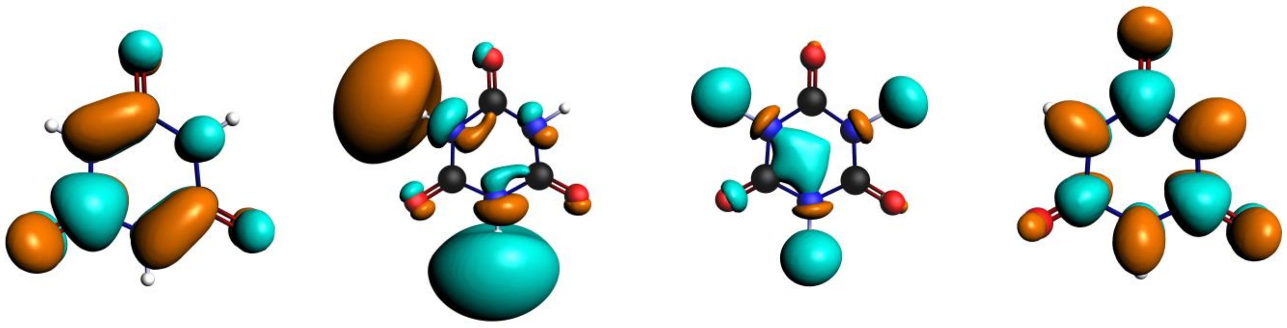

$5b_1$ (LUMO) **$9b_2$** **$14a_1$** **$7b_1$**

Figure S6. 3D plots of selected final MOs of CA relative to the O1s DFT-TP core excitation calculations. Displayed isosurface corresponds to ±0.03 e $^{1/2}$ $a_0^{-3/2}$ value for $5b_1$ and $7b_1$ MOs and to ±0.025 e $^{1/2}$ $a_0^{-3/2}$ value for $9b_2$ and $14a_1$ MOs.

Table S7. Peak assignment of the O1s NEXAFS spectrum of H-B model: DFT-TP excitations energies (eV) and oscillator strengths *f* of the main below edge transitions.
.

Peak	E(eV)	[a]*f*x10^2	Assignment, main character of the final MO
A	532.73	2.33	25 b_1 (LUMO)/ π*(C=O) + π*(C-N)
B	536.61	0.15	34 b_1/ π*(C=O) +π*(C-N) / Rydberg
	536.69	0.13	35 b_1/Rydberg

[a] Only transitions with *f*x10^2≥0.10 are reported

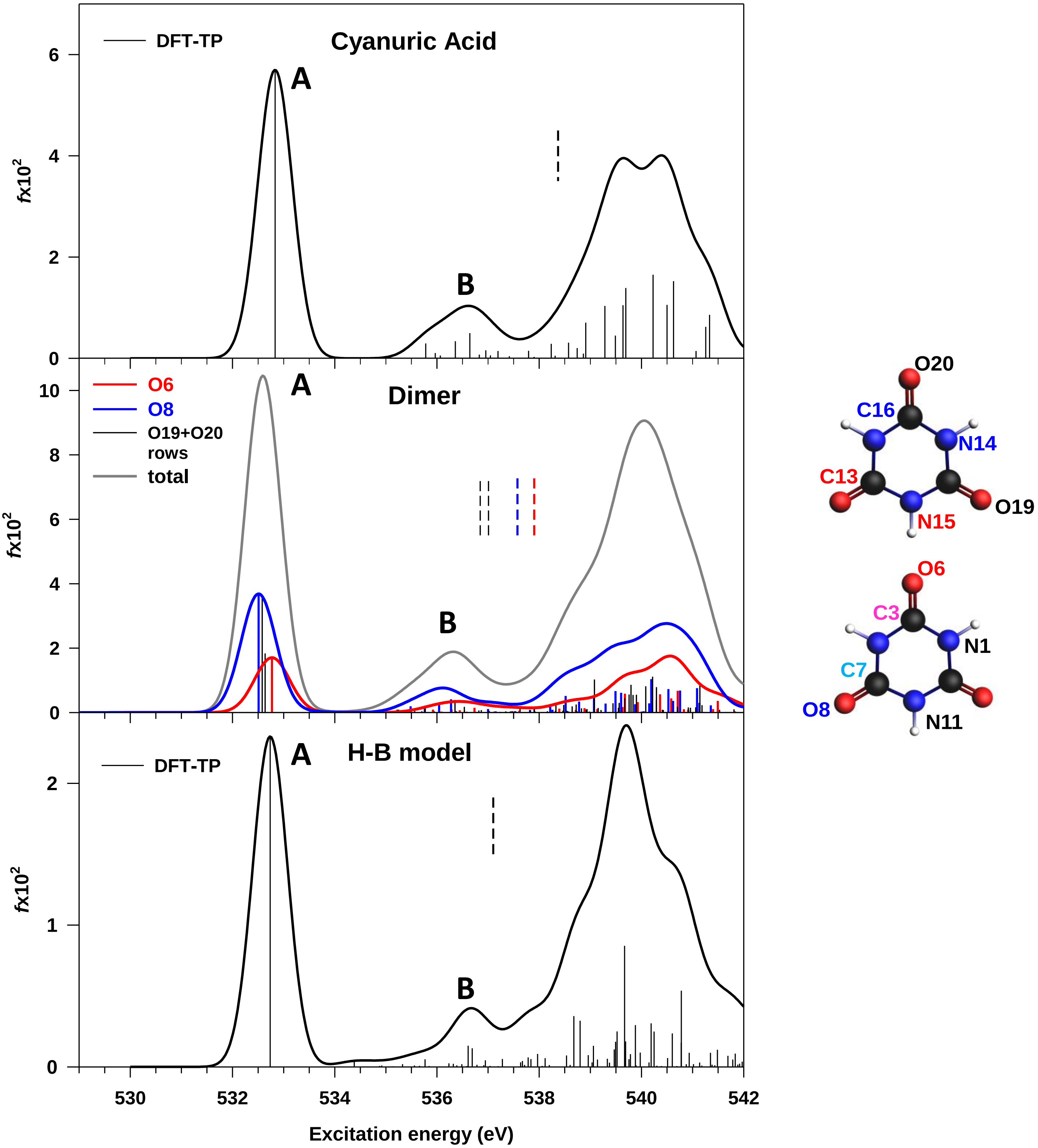

Figure S7. DFT-TP calculated O K-edge NEXAFS spectra of CA (upper panel), CA dimer (middle panel) and H-B model (lower panel). The partial contributions of the non-equivalent O atoms to the total spectrum of the dimer are also highlighted with different colours. The stick spectra are broadened by using a Gaussian line shape with FWHM = 0.8 eV. The calculated ΔSCF O1s IPs are indicated with vertical dashed lines. The chemical structure of the dimer is reported on the right side.

The O1s NEXAFS spectrum of the dimer does not add significant information, as it emerges from Figure S7 of the SI, which compare the DFT-TP result for CA, dimer and H-B model. The DFT-TP results for the dimer are collected in Table S8 of the SI. The spectral variations of the dimer with respect to the CA spectrum are negligible and also the nature of the transitions contributing to the A and B structures is preserved. The partial spectral contributions of the non- equivalent O sites of the dimer are reported, in particular the profiles of the O6 and O8 sites belonging to the (H-acceptor) ring of the dimer are highlighted. The O8 site is expected to be similar to the O site of the monomer while O6 is the O(H-acceptor) site involved in the H-bond interaction. Actually only minor variations affect the profile of the O6 site compared to the O8 profile and this behaviour confirms that the O (H-acceptor) site is less perturbed by the H-bond formation compared to the N (H-donor) site.

Table S8. Peak assignment of the C1s NEXAFS spectrum of gas phase CA: DFT-TP excitation energies (eV) and oscillator strengths *f* of the main below edge transitions.

Peak	E(eV)	[a] E (eV)	[b]fx10^2	Assignment, main character of the final MO
**A**	290.56	289.51	18.6	5 b_1 (LUMO)/ π*(C=O) + π*(C-N)
**B**	294.44	293.39	1.00	6 b_1/π*(C=O) + π*(C-N)
	294.80	293.75	1.55	7 b_1/π*(C=O) + π*(C-N)
	295.15	294.10	0.53	10 b_2/σ*(C-N) + σ* (N-H)/Rydberg
**C**	295.86	294.81	0.40	19 a_1/σ*(N-H) /Rydberg
	295.91	294.86	1.00	20 a_1/Rydberg
	296.08	295.03	0.72	12b_2/σ*(C-N) + σ* (N-H)/Rydberg
Higher excitations	296.59	295.54	1.61	13b_2/Rydberg
	296.69	295.64	1.75	22a_1/Rydberg
	297.01	295.96	3.48	14b_2/Rydberg

[a] Calculated excitation energies shifted by −1.05 eV to match the first experimental peak.
[b] Only transitions with fx10^2≥0.50 are reported.

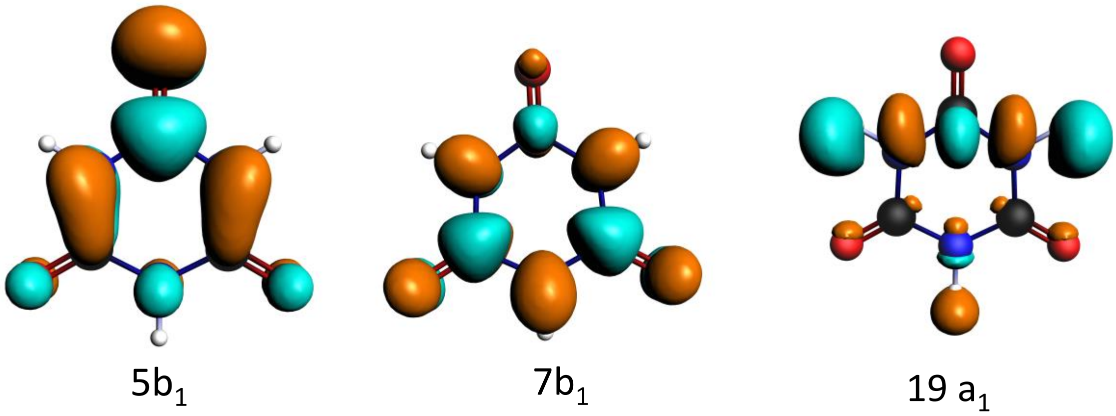

Figure S8. 3D plots of selected final MOs of CA relative to the C1s DFT-TP core excitation calculations. Displayed isosurface corresponds to ±0.03 $e^{1/2}$ $a_0^{-3/2}$ value for $5b_1$ and $7b_1$ MOs and to ±0.025 $e^{1/2}$ $a_0^{-3/2}$ value for $19a_1$ MOs.

Table S9. Peak assignment of the C1s NEXAFS spectrum of H-B model: DFT-TP excitations energies (eV) and oscillator strengths *f* of the main below edge transitions.

Peak	E(eV)	fx10^2	Assignment, main character of the final MO
A	290.32	9.19	25 b_1 (LUMO)/π*(C=O) + π*(C-N)
B	294.62	0.80	33 b_1/ π*(C-N) + π*(C=O) /Rydberg
	294.66	0.40	34 b_1/ π*(C-N) + π*(C=O) /Rydberg

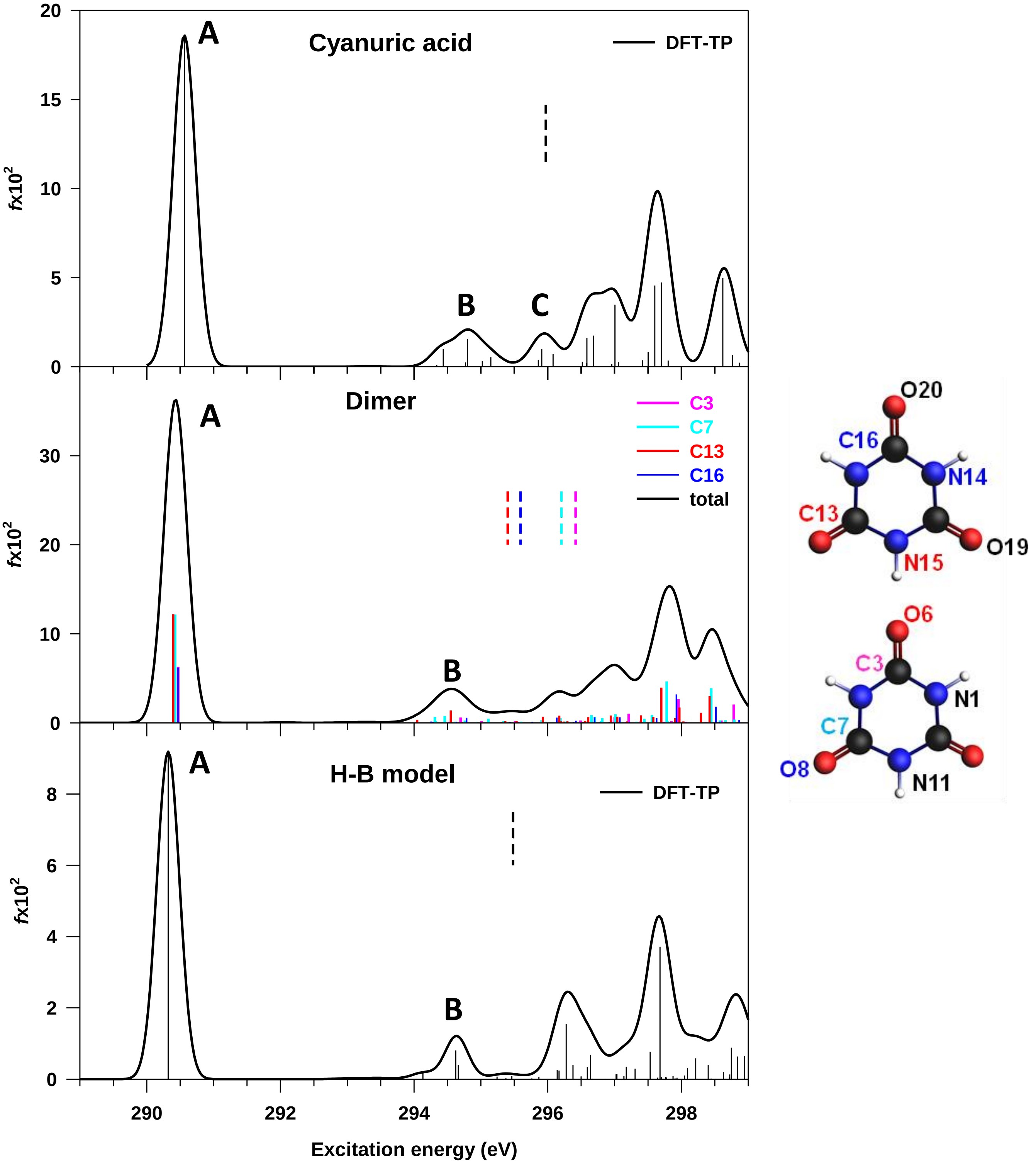

Figure S9. DFT-TP calculated C K-edge NEXAFS spectra of CA (upper panel), CA dimer (middle panel) and H-B model (lower panel). The partial contributions of the non-equivalent C atoms to the total spectrum of the dimer are also highlighted with different colours. The stick spectra are broadened by using a Gaussian line shape with FWHM = 0.4 eV. The calculated ΔSCF O1s IPs are indicated with vertical dashed lines. The chemical structure of the dimer is reported on the right side.
